# Pathway-specific polygenic scores substantially increase the discovery of gene-adiposity interactions impacting liver biomarkers

**DOI:** 10.1016/j.xhgg.2025.100515

**Published:** 2025-09-11

**Authors:** Kenneth E. Westerman, Daniel I. Chasman, W. James Gauderman, Arun Durvasula

**Affiliations:** 1 Clinical and Translational Epidemiology Unit, Massachusetts General Hospital, Boston, MA, USA; 2 Department of Medicine, Harvard Medical School, Boston, MA, USA; 3 Programs in Metabolism and Medical and Population Genetics, Broad Institute of MIT and Harvard, Cambridge, MA, USA; 4 Division of Preventive Medicine, Brigham and Women’s Hospital, Boston, MA, USA; 5 Division of Biostatistics, Department of Population and Public Health Sciences, Keck School of Medicine, University of Southern California, Los Angeles, CA, USA; 6 Division of Epidemiology, Department of Population and Public Health Sciences, Keck School of Medicine, University of Southern California, Los Angeles, CA, USA; 7 Center for Genetic Epidemiology, Department of Population and Public Health Sciences, Keck School of Medicine, University of Southern California, Los Angeles, CA, USA; 8 Department of Quantitative and Computational Biology, University of Southern California, Los Angeles, CA, USA

## Abstract

Polygenic scores (PGSs) are appealing for detecting gene-environment interactions due to the aggregation of genetic effects and reduced multiple testing burden compared to single-variant genome-wide interaction studies (GWISs). However, standard PGSs reflect many different biological mechanisms, limiting interpretation and potentially diluting pathway-specific interaction signals. Previous work has uncovered a significant genome-wide PGS×Adiposity signal impacting liver function, but there is an opportunity for additional and more interpretable discoveries. Here, we leveraged pathway-specific polygenic scores (pPGSs) to discover mechanism-specific gene-adiposity interactions. We tested for body mass index (BMI) interactions impacting three liver-related biomarkers (ALT, AST, and GGT) using (1) a standard, genome-wide PGS, (2) an array of pPGSs containing variant subsets derived from KEGG pathways, and (3) a GWIS. For ALT, we identified 49 significant pPGS×BMI interactions at a Bonferroni corrected *p* < 2.7 × 10^−4^, 80% of which were not explained by genes close to the 8 loci found in the associated GWIS. Across all biomarkers, we found interactions with 83 unique pPGSs. We tested alternate pathway collections (hallmark, KEGG Medicus), finding that the choice of pathway collection strongly impacts discovery. Our findings reinforced known biology (e.g., glycerolipid metabolism and hepatic lipid export affecting ALT release) and captured additional phenomena (e.g., actin cytoskeleton remodeling-associated variants alter the liver’s robustness to lipid mechanical stress and thus GGT release). These results support the use of pPGSs for well powered and interpretable discovery of pPGS×E interactions with adiposity-related exposures for liver biomarkers and motivate future studies using a broader collection of exposures and outcomes.

## Teaser

The authors describe a strategy for using pathway-specific polygenic scores to increase the detection and interpretability of gene-environment interactions, with an applied analysis uncovering genetic modification of the relationship between adiposity and liver stress.

## Main text

Recent work has shown that gene-environment interactions (G×E) have a substantial impact on complex disease and trait variation.^1^^,^^2^^,^^3^^,^^4^^,^^5^^,^^6^ However, discovery and interpretation of single-variant G×E via genome-wide interaction studies (GWISs) is challenging due to the large number of hypothesis tests that exacerbate the intrinsically low power of interaction tests.^7^ Meanwhile, polygenic approaches, such as genome-wide polygenic score-by-E tests (gwPGS×E), may lose power by leveraging a strong and rarely satisfied assumption that main and interaction effects are proportional.^6^^,^^8^^,^^9^ Recently, Durvasula and Price^6^ proposed a conceptual G×E model in which environmental exposures modify genetic effects on specific pathways rather than across the entire genome, motivating G×E analyses on the pathway level as a compromise between the specificity of GWISs and the power of gwPGS×E tests.

Chasman et al.^10^ provided early proof-of-concept for this approach by conducting a data-driven clustering of disease-associated genetic loci followed by cluster-specific G×E testing, focusing on cardiovascular diseases and type 2 diabetes.^11^ Gauderman et al. focused on pre-established pathway annotations, showing by simulation that the use of pathway-based pPGS×E can lead to substantial increases in power over testing gwPGS×E. They also applied the approach to colorectal cancer, aggregating genome-wide significant SNPs within annotated pathways and identifying significant pPGS×E that were not detected by gwPGS×E analysis.^11^ These studies underscore the potential of pathway-specific polygenic score (pPGS) approaches for increasing power to detect G×E interactions, but it remains unclear how these pPGS interactions compare to the equivalent genome-wide, variant-specific interaction study and whether the results differ substantially by choice of pathway database.

Leveraging the annotation-based pathway approach, we sought to explore genetic modification of the relationship between adiposity (as measured by body mass index [BMI]) and liver stress biomarkers. Our prior work demonstrated the presence of a polygenic signature modifying the relationship between BMI and cardiometabolic risk factors.^12^ There was a particularly strong signature for liver-related biomarkers, which are indicative of multiple types of liver damage (see supplemental methods). We showed preliminary evidence that interactions with BMI reflected a mechanism-specific subset of the overall genetic architecture of these biomarkers. Here, we explore this biological question further as an applied setting to test the incorporation of existing biological annotations and pPGSs into an interaction testing framework.

**Figure 1:**
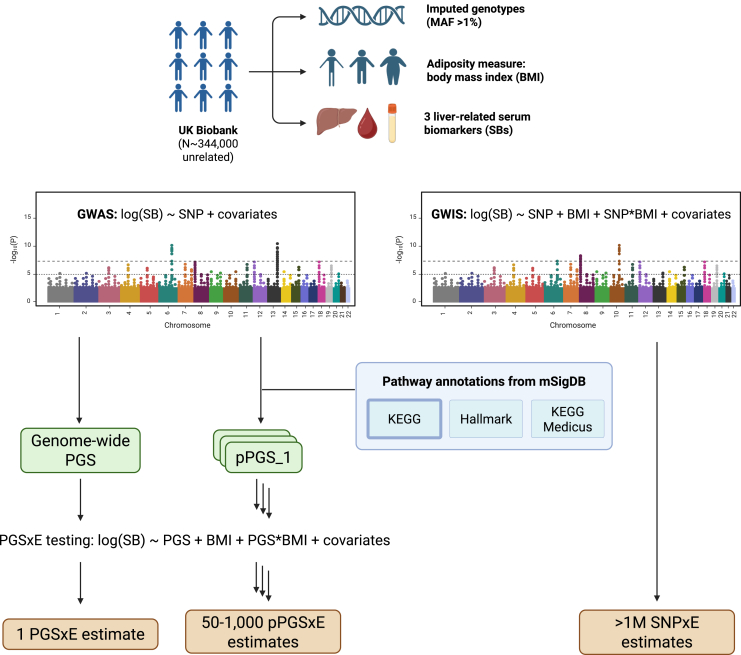
Pipeline for multi-scale gene-by-body mass index interaction (G×BMI) testing carried out in this study

Our analysis pipeline is summarized in Figure 1. We analyzed individual-level data for 344,000 unrelated participants of European ancestry from the UK Biobank (UKB). We performed a standard GWAS for each of three log-transformed liver-related biomarkers (alanine aminotransferase [ALT], aspartate aminotransferase [AST], and gamma-glutamyl transferase [GGT]) in the entire UKB dataset. We used these summary statistics to create a genome-wide PGS (gwPGS) as well as a series of pPGSs based on KEGG (Kyoto Encyclopedia of Genes and Genomes) pathway annotations. We then tested each PGS for interaction with BMI. For comparison to the PGS-based interaction tests, we performed a GWIS for each biomarker with BMI as the exposure.

We first tested for the interaction between genome-wide PGS and BMI in a regression including a main effect for the gwPGS and adjusting for age, age^2^, sex, age×sex, 10 genomic principal components (gPCs), and 10 BMI×gPC product terms. We used a pruning and thresholding (P&T) approach to train the gwPGSs, retaining between 4612 and 6164 SNPs at a *p* value threshold of 0.001 (across the three biomarkers). We identified strong gwPGS×E interactions for all three liver biomarkers. For ALT, AST, and GGT, these interaction effect sizes were 0.035 SD_log(ALT)_/SD_PGS_/SD_BMI_ (SD, standard deviation; *p* = 1.7 × 10^−133^), 0.028 (*p* = 7.8 × 10^−74^), and 0.022 (*p* = 3.7 × 10^−58^), respectively. These results are concordant with our recent analyses of PGS×BMI for liver biomarkers,^12^ and all represent patterns in which higher PGS values magnify the positive BMI-biomarker relationship. We note that there is no expected bias due to training a standard PGS and testing it for interaction in the same dataset, as demonstrated by Gauderman and colleagues.^11^

**Figure 2:**
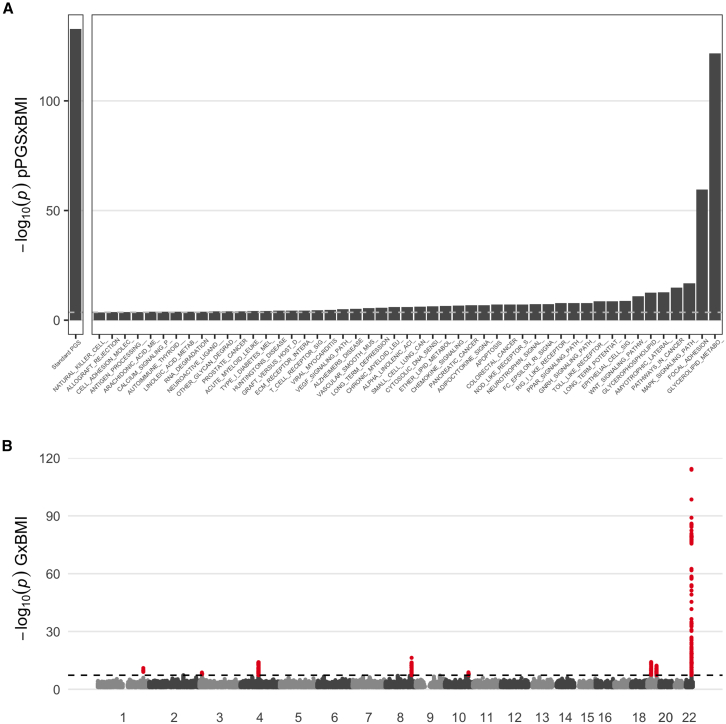
G×Es shape the relationship between adiposity and alanine aminotransferase (ALT) (A) PGS×E regression *p* values for the gwPGS (left) and each significant pPGS (right). *x* axis pathway labels are truncated for the purpose of visualization. (B) Manhattan plot shows SNP-specific *p* values as a function of genomic position. Results for AST and GGT are provided in Figures S2 and S3. Numeric results for all biomarkers are provided in Tables S1 and S2.

Next, we investigated the discovery enabled by pathway-level testing. We assigned SNPs to genes based on physical distance to the closest annotated gene (−2 kb to +1 kb from the gene transcription start site and transcription end site, respectively) and assigned genes to pathways using gene sets from the mSigDB database, focusing on the KEGG gene-to-pathway mappings as the primary pathway group of interest (Figure S1). We calculated the pPGS for each pathway using a *p*-value-based P&T approach as implemented in the PRSet program,^13^ using a *p* value threshold of 0.001. We tested for interactions between pPGS and BMI using regression models analogous to those used for the standard gwPGS above, assigning significance based on a Bonferroni correction for the 186 total pathways tested. We identified a substantial number of significant pPGS×E for all three biomarkers (Figure 2B for ALT; full set of results in Table S1). First, we found 49 significant pPGS×E interactions for ALT. Though no single pPGS reached the same degree of significance as the gwPGS, interactions were highly significant for multiple pathways, including glycerolipid metabolism (*p* = 2.0 × 10^−122^) and focal adhesion (*p* = 2.9 × 10^−60^) (Figure 2A). The glycerolipid metabolism association is concordant with previous work showing a relationship between glycerolipid metabolism and obesity^14^ as well as obesity and liver biomarkers.^15^ Results for AST were similar, with the same top two pathways and 37 significant pathways in total (Figure S2). Notably, for AST, the same glycerolipid metabolism pathway reached a greater degree of significance than the gwPGS (*p* = 2.1 × 10^−133^), highlighting the value of the pPGS in improving power by prioritizing relevant genomic regions. GGT analyses resulted in 31 significant pathways, with a notably different pattern of significance across pathways compared to ALT and AST, suggesting a different architecture of G×Es (Figure S3). Here, the top pathways included regulation of actin cytoskeleton, pathways in cancer, peroxisome, and glutathione metabolism. The collection of all pPGS interactions added substantial signal in aggregate: models that included all pPGS×BMI interactions effects fit the data significantly better than models with only gwPGS×BMI interaction effects (ALT *p* = 5 × 10^−66^, AST *p* = 4 × 10^−99^, GGT *p* = 1 × 10^−7^; see supplemental methods for description of likelihood ratio tests). Taken together, these results show that pPGS×E analysis can break down genome-wide gwPGS×E signals into biologically interpretable results.

Next, we performed a single-variant GWIS for each biomarker using analogous regression models to that for the gwPGS and pPGS, additionally using robust standard errors and applying a genome-wide significance threshold of 5 × 10^−8^. We found 8 genome-wide significant loci for each of ALT and AST (7 of these were overlapping) and 2 for GGT (Figures 1B, S2, and S3; Table S2) after a simple distance-based pruning ( ±500 kb from lead variants). The strongest locus for both ALT and AST mapped to the *PNPLA3* gene on chromosome 22, a well-known locus affecting liver disease and metabolic traits,^16^ whose strong main effect on ALT and AST has previously been shown to be modified by BMI in UKB.^12^ All 8 genome-wide significant GWIS loci also achieved significance in the associated GWAS for liver biomarkers, in analysis examining solely main effects.

Next, we asked how many of the pPGS×E results overlapped with biological pathways that could have been discovered via the GWIS approach. We annotated pPGS×E tests as “explained” if there was at least one GWIS lead variant near any gene in that pathway set (within 100 kb of the TSS based on Ensembl GRCh37 v75). For ALT, AST, and GGT, we found that 39 (80%), 34 (92%), and 22 (71%) pathway interactions were unexplained by significant GWIS loci (Table 1), respectively. These findings indicate that the majority of pPGS×E interactions point to pathways that would not have been found using a single-variant approach. When testing for enrichment of GWIS signal using the MAGMA tool and the same set of variant-gene-pathway annotations, we found that results were significantly correlated with those from the pPGS×E approach (r_Spearman_ = 0.25 for −log_10_(*p*) values, *p* = 2.8 × 10^−9^) but systematically less significant (Figure S4). In addition, all pathways highlighted by this GWIS enrichment approach were discovered by the pPGS×E approach. The two strongest pathways from the pPGS×E approach for ALT and AST (glycerolipid metabolism and focal adhesion) showed minimal signal in the enrichment-based approach (*p* > 0.05 for both). Taken together, these results highlight the increased power of pPGS×E to discover G×E interactions over GWIS.

**Table 1:** Overlap between significant findings from the single-variant GWIS and pPGS approaches

Biomarker	# GWIS loci	# pPGS×BMI	# “Unexplained” pPGS×BMI pathways	Fraction “unexplained” pPGS×BMI pathways
ALT	8	49	39	80%
AST	8	37	34	92%
GGT	2	31	22	71%

To understand the impact of the choice of pathway database (i.e., gene-to-pathway mapping) on our results, we reran the same analysis pipeline using two additional pathway collections from mSigDB: hallmark pathways and KEGG Medicus (see Figure S1 for metadata describing these collections). Over all biomarkers, these collections resulted in 83 (KEGG), 36 (hallmark), and 67 (KEGG Medicus) significant pPGS×BMI interactions. The comparison of KEGG Medicus, an expansion of KEGG to include disease- and drug-related annotations, to the primary KEGG “legacy” collection indicates the importance of pathway collection choice: fewer significant pathways were uncovered despite a much larger number of available pathways (658 for Medicus versus 186 for legacy) due to the specific biological pathways represented and the differential multiple testing burden.

We used a series of secondary analyses to address potential concerns. First, we chose a single P&T *p* value threshold of 0.001 based on optimized main effect PGS thresholds from a prior UKB analysis.^12^ We confirmed that this choice did not substantially impact the pPGS×E results: interaction effects based on pPGS using a P&T threshold of 5 × 10^−8^ showed minimal difference in significance (Figure S5). Second, we calculated an adjusted (or “effective”) number of pathways discovered to account for the fact that some pathways are correlated (see supplemental methods for details on the PCA-based method). This resulted in a reduction of significant KEGG pathways from 49 to 9.6 effective pathways for ALT, 21 to 11.1 for AST, and 10 to 2.8 for GGT. Third, we verified that the uncovered interactions were not due solely to the pPGS tracking with the genetics of BMI: interaction estimates changed minimally when adjusting models for a genome-wide PGS for BMI (PGS_BMI_) or, additionally, a PGS_BMI_×BMI product term (Figure S6). Finally, we confirmed that developing pPGS and testing for interactions in the same sample set does not induce bias, which was previously studied using simulations.^11^ We repeated our pPGS×E interaction tests in a 20% subset of the UKB and found similar results when using pPGS developed in the rest of the dataset as opposed to the full dataset (Figure S7; supplemental methods).

We leveraged pathway annotations and the UK Biobank with complex trait, exposure, and genetic data to identify gene-environment interactions acting on specific pathways. We found 83 significant KEGG pathway pPGS×BMI interactions across three liver biomarkers, pointing to specific biological processes modifying the relationship between adiposity and liver health.

The highly significant glycerolipid pathway finding for ALT and AST captures an interaction that we and others have described: genetic effects on this pathway’s function alter the liver’s ability to relieve adiposity-associated lipid buildup.^17^ The top hit for GGT was regulation of actin cytoskeleton; this may point to the importance of cytoskeletal and mechanical integrity for maintaining proper bile flow, whose deterioration impacts GGT more than ALT or AST.^18^ Obesity-associated lipid buildup can increase mechanical stress in hepatocytes^19^ and disrupt the bile canaliculus network,^20^ with potential modification of this effect by genetic effects on cytoskeletal integrity. While the preceding pathways have been studied in the context of liver health, additional highly significant pathways from the pPGS interaction approach may point to additional mechanisms of obesity-related liver pathogenesis. For example, the disease-associated amyotrophic lateral sclerosis (ALS) pathway was one of the strongest for ALT. While ALS is a motor neuron disease not classically associated with liver dysfunction, patients are highly enriched for hepatic steatosis, possibly via mitochondrial and endoplasmic reticulum stress pathways,^21^ suggesting directions for further study.

Our study represents an advance over previous studies investigating pathway-specific polygenic scores for interaction. Our approach based on *a priori* pathway annotations increases the interpretability of our pPGS×E results compared to data-driven approaches. Leveraging these strengths, we show that pPGS×E testing enables increased discovery of gene-environment interactions and produces interactions that (1) are mostly unexplained by the associated variant-specific GWIS and (2) can be stronger than the associated gwPGS×E test. Furthermore, we show that the choice of pathway annotation (variant to gene to pathway) has a substantial impact on pPGS×E results and more comprehensive exploration of additional pathway collections is likely to further increase the number of interactions uncovered.

Our study has several limitations. First, we used a simple distance-based method for variant-to-gene mapping and a limited set of pathway annotations. Future work could apply more sophisticated and wide-ranging methods making use of functional genomic data to assign SNPs to genes and ultimately pathways. Second, we study a limited subset of biomarkers and only one exposure. Our methodological conclusions may not hold universally across all biomarkers and exposures. Third, we did not account for correlated pathway annotations, which makes our stringent Bonferroni correction for the total number of pathways conservative. Future work could model the correlated tests to increase statistical power. Despite these limitations, our work highlights pathway-specific PGS×E testing as a powerful way to discover G×Es.

## Acknowledgments

K.E.W. was supported by National Institutes of Health K01DK133637. A.D. was supported by NIH R35GM160467.

## Declaration of interests

The authors declare no competing interests.

## Supplemental information

Data S1. Tables S1 and S2

Document S2. Article plus supplemental information
