# Supplementary material for: Pathway-specific polygenic scores substantially increase the discovery of gene-adiposity interactions impacting liver biomarkers
Source: HGG Adv. 2025 Sep 11;7(1):100515. doi: 10.1016/j.xhgg.2025.100515 (PMC12508838; doi:10.1016/j.xhgg.2025.100515)
Supplement: Document S1. Figures S1–S7 and supplemental methods [file mmc1.pdf]

HGGA, Volume 7

Supplemental information

Pathway-specific polygenic scores substantially increase the discovery of gene-adiposity interactions impacting liver biomarkers

Kenneth E. Westerman, Daniel I. Chasman, W. James Gauderman, and Arun Durvasula

Supplementary Methods

UK Biobank data

We used data from the large, prospective UK Biobank cohort in all analyses[1]. This research was conducted using the UK Biobank resource under application no. 277892 and Not Human Subjects Research determination NHSR-4298 at the Broad Institute of MIT and Harvard. Genotyping, imputation, and initial quality control on the genetic dataset have been described previously[2]. Work was conducted on genetic data release version 3, with imputation to both Haplotype Reference Consortium[3] and 1000 Genomes Project (1KGP)[4]. Ultimately, analysis was performed on a set of unrelated individuals, defined as the set of individuals whose genomes were included in centrally performed genetic principal components analysis. As described in prior related work[5] , we excluded individuals that had withdrawn consent by the time of analysis excluded as well as those with diabetes, coronary heart disease, cirrhosis, end-stage renal disease, cancer diagnosis within one year prior to their assessment center visit, or who were pregnant within one year of the assessment center visit.

Body mass index (BMI; kg/m^2) was collected from assessment center anthropometric measurements. Serum biomarker values were measured in blood samples collected at the baseline visit (details available at: https://biobank.ctsu.ox.ac.uk/crystal/crystal/docs/serum_biochemistry.pdf).

Liver biomarkers

We focused our analyses on three biomarkers for liver function: alanine aminotransferase (ALT), aspartate aminotransferase (AST), and gamma-glutamyl transferase (GGT). These biomarkers are used to diagnose liver diseases, including non-alcoholic fatty liver disease[6]. ALT is the most liver-specific of these biomarkers, with elevation indicating hepatocellular injury[7]. AST is also elevated with hepatocellular injury, but is more closely tied to alcohol-related steatohepatitis and can also suggest other sources of physiological stress, such as myopathy[7]. GGT elevation, in contrast, is often indicative of a cholestatic pattern involving bile duct obstruction[7]. Due to their skewed distribution, we log-transformed each biomarker prior to analysis. We avoid issues of apparent interactions caused by scale effects for binary outcomes by focusing on biomarker levels rather than disease status[8].

Genome-wide studies

A genome-wide association study (GWAS) and genome-wide interaction study (GWIS) was performed for each log-transformed biomarker. The GWAS used a basic linear model corrected for covariates:

$$Y = G + C,$$

We adjusted for the following covariates: age, age^2, sex, and ten genetic PCs. We used model-based standard errors and included only variants with minor allele frequency >1% and imputation quality INFO score >0.5 (approximately 9,891,000 variants in total). The GWIS model added an environmental exposure and its product term with G:

$$Y = G + E + G \times E + C,$$

In the GWIS, we additionally adjusted for an E×gPC product term for each gPC[9] and used robust standard errors. All genome-wide studies were conducted using GEM v1.5.2[10].

Pathway annotations and polygenic score generation

We generated PGS from GWAS summary statistics using the PRSet program[11], which builds on PRSice-2[12]. PRSet both computes pathway-specific PGS weights (using the P&T approach along with an LD reference panel) and calculates scores for the input UKB dataset as a linear combination of genotypes based on those weights. As input parameters governing the behavior of the P&T algorithm, we used p-value thresholds of both 0.001 and $5x10^{-8}$, a clumping radius of 1MB, an r^2 threshold of 0.1, and an LD reference panel consisting of a random 20,000 individuals from the UKB. In all analyses, we considered only autosomal variants and removed ambiguous variants (A/T or C/G) during PGS development.

To generate pPGS, we additionally obtained pathway annotations from mSigDB[13]. We used three sets of pathway collections: 1) KEGG[14], 2) Hallmark[13], and 3) KEGG Medicus[14]. PRSet assigns variants to pathways via physical proximity to constituent genes and subsequently conducts a separate P&T procedure for each pathway. For this variant to gene mapping, we included variants within a boundary of 2kb upstream (5') and 1kb downstream (3') of the gene transcription start site and end site, respectively. By default, PRSet also generates a gwPGS using the same P&T procedure but including all available variants, resulting in gwPGS containing 4,612, 4,894, and 6,164 variants for ALT, AST, and GGT, respectively.

Polygenic score by environment testing

We tested for interactions using linear regression of the form:

$$Y = PGS + E + PGS \times E + C,$$

where Y is the trait value, PGS is the polygenic score for the trait, E is the environment variable, and C is a set of covariates. For pathway PGS, we used the following linear regression for a single pathway:

$$Y = pPGS_i + E + pPGS_i \times E + C,$$

Where $i \in P$ indexes the pathway in the set of pathways P (see Pathway annotations and polygenic score generation). For all analyses, we correct for the same covariates as in the GWIS: age, age^2, sex, ten genetic PCs, and an E×gPC product term for each gPC[9]. We used the p-value associated with the interaction term to assess significance. To account for multiple testing in the pathway specific PGS, we used a Bonferroni corrected threshold of 0.05 divided by the number of pathways tested. All individual-level data preprocessing and regression analysis after PGS generation was performed using R v4.1 and 4.2[15] except where otherwise noted.

We used likelihood ratio tests to understand whether the cumulative contribution of many pPGS×BMI interactions explained significantly more variance than the gwPGS×BMI interaction alone. The restricted model included all covariates from the basic pPGS×BMI interaction tests (see above) as well as a main effect for the gwPGS, main effects for each pPGS, and an interaction term for the gwPGS. The full model added interaction terms for each of the pPGS. We tested for significance of the additional variance explained by the full model using the *lmtest::lrtest()* function.

To enable us to a report a number of discoveries that accounted for the substantial correlation between pPGS, we calculated an "effective" number of pathways discovered for each biomarker

using a previously described method[16]. Briefly, we subsetted a rectangular matrix of individual-level pPGS values to include only those that were significant for the biomarker of interest, then performed principal components analysis (*prcomp* function with standardized variables). The number of effective biomarkers was then calculated from the principal component variances λ (equal to the eigenvalues of the biomarker covariance matrix) as $N_{BM,eff} = \frac{\left(\sum_{k=1}^{p} \lambda_k\right)^2}{\sum_{k=1}^{p} \lambda_k^2}$.

Sensitivity analyses

To understand whether the pPGS interactions were due to their prediction of BMI (rather than liver biomarkers), we also generated a genome-wide PGS for BMI (as the outcome phenotype). We followed the same procedure as used for each liver biomarker, selecting only the whole-genome score for downstream sensitivity analysis.

To evaluate the impact of our "in sample" training and testing procedure, we performed an additional analysis mirroring the original, but using separate training (70%), tuning (10%), and testing (20%) data subsets. Unlike the original analysis, this enabled the selection of an optimal *p*-value threshold for each pathway based on prediction performance in the tuning set (thresholds in {5×10^{-8}, 5×10^{-7}, 5×10^{-6}, …, 0.05}). These optimized pPGS were then tested for interaction with BMI in the held-out testing set. Importantly, the results from this analysis ("80/20") were compared with those using pPGS from the primary analysis but tested in the same 20% of participants ("100/20"). We note that in this comparison, beyond any influence of sample overlap, the 100/20 analysis benefits slightly from having conducted the GWAS in a larger sample.

Supplementary References

1. Sudlow, C. *et al.* UK biobank: an open access resource for identifying the causes of a wide range of complex diseases of middle and old age. *PLoS Med.* **12**, e1001779 (2015).
2. Bycroft, C. *et al.* The UK Biobank resource with deep phenotyping and genomic data. *Nature* **562**, 203–209 (2018).
3. McCarthy, S. *et al.* A reference panel of 64,976 haplotypes for genotype imputation. *Nat. Genet.* **48**, 1279–1283 (2016).
4. Byrska-Bishop, M. *et al.* High-coverage whole-genome sequencing of the expanded 1000 Genomes Project cohort including 602 trios. *Cell* **185**, 3426-3440.e19 (2022).
5. Westerman, K. E., Gervis, J. E., O'Connor, L. J., Udler, M. S. & Manning, A. K. Polygenic scores capture genetic modification of the adiposity-cardiometabolic risk factor relationship. *medRxiv* 2025.04.09.25324066 (2025) doi:10.1101/2025.04.09.25324066.
6. Sanyal, A. J. *et al.* Diagnostic performance of circulating biomarkers for non-alcoholic steatohepatitis. *Nat. Med.* **29**, 2656–2664 (2023).
7. Lala, V., Zubair, M. & Minter, D. A. Liver function tests. in *StatPearls* (StatPearls Publishing, Treasure Island (FL), 2025).
8. Durvasula, A. & Price, A. L. Distinct explanations underlie gene-environment interactions in the UK Biobank. *Am. J. Hum. Genet.* **112**, 644–658 (2025).
9. Keller, M. C. Gene × environment interaction studies have not properly controlled for potential confounders: the problem and the (simple) solution. *Biol. Psychiatry* **75**, 18–24 (2014).

10. Westerman, K. E. *et al.* GEM: scalable and flexible gene-environment interaction analysis in millions of samples. *Bioinformatics* **37**, 3514–3520 (2021).
11. Choi, S. W. *et al.* PRSet: Pathway-based polygenic risk score analyses and software. *PLoS Genet.* **19**, e1010624 (2023).
12. Choi, S. W. & O'Reilly, P. F. PRSice-2: Polygenic Risk Score software for biobank-scale data. *Gigascience* **8**, giz082 (2019).
13. Liberzon, A. *et al.* The Molecular Signatures Database (MSigDB) hallmark gene set collection. *Cell Syst.* **1**, 417–425 (2015).
14. Kanehisa, M., Furumichi, M., Tanabe, M., Sato, Y. & Morishima, K. KEGG: new perspectives on genomes, pathways, diseases and drugs. *Nucleic Acids Res.* **45**, D353–D361 (2017).
15. R Core Team. R: A language and environment for statistical computing. *R Foundation for Statistical Computing, Vienna, Austria. URL https://www.r-project.org/* (2022).
16. Wang, H. *et al.* Genotype-by-environment interactions inferred from genetic effects on phenotypic variability in the UK Biobank. *Sci Adv* **5**, eaaw3538 (2019).

Supplementary Figures

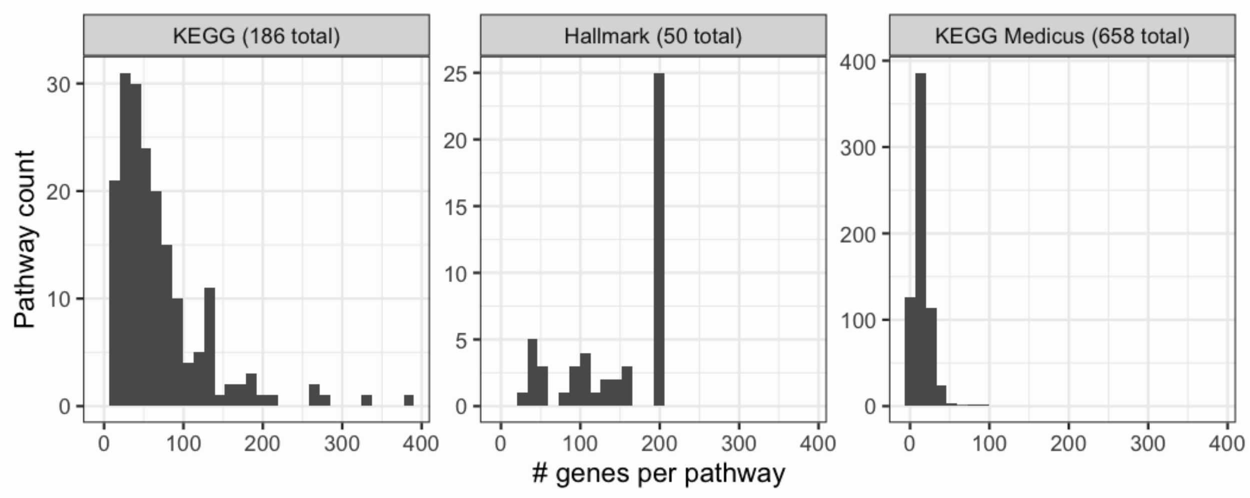

Supplementary Figure S1. Summary of selected pathway annotations from mSigDB. For each panel (corresponding to pathway groups, along with their total number of pathways), the histogram displays counts of pathways (*y*-axis) having a given number of constituent genes (*x*-axis).

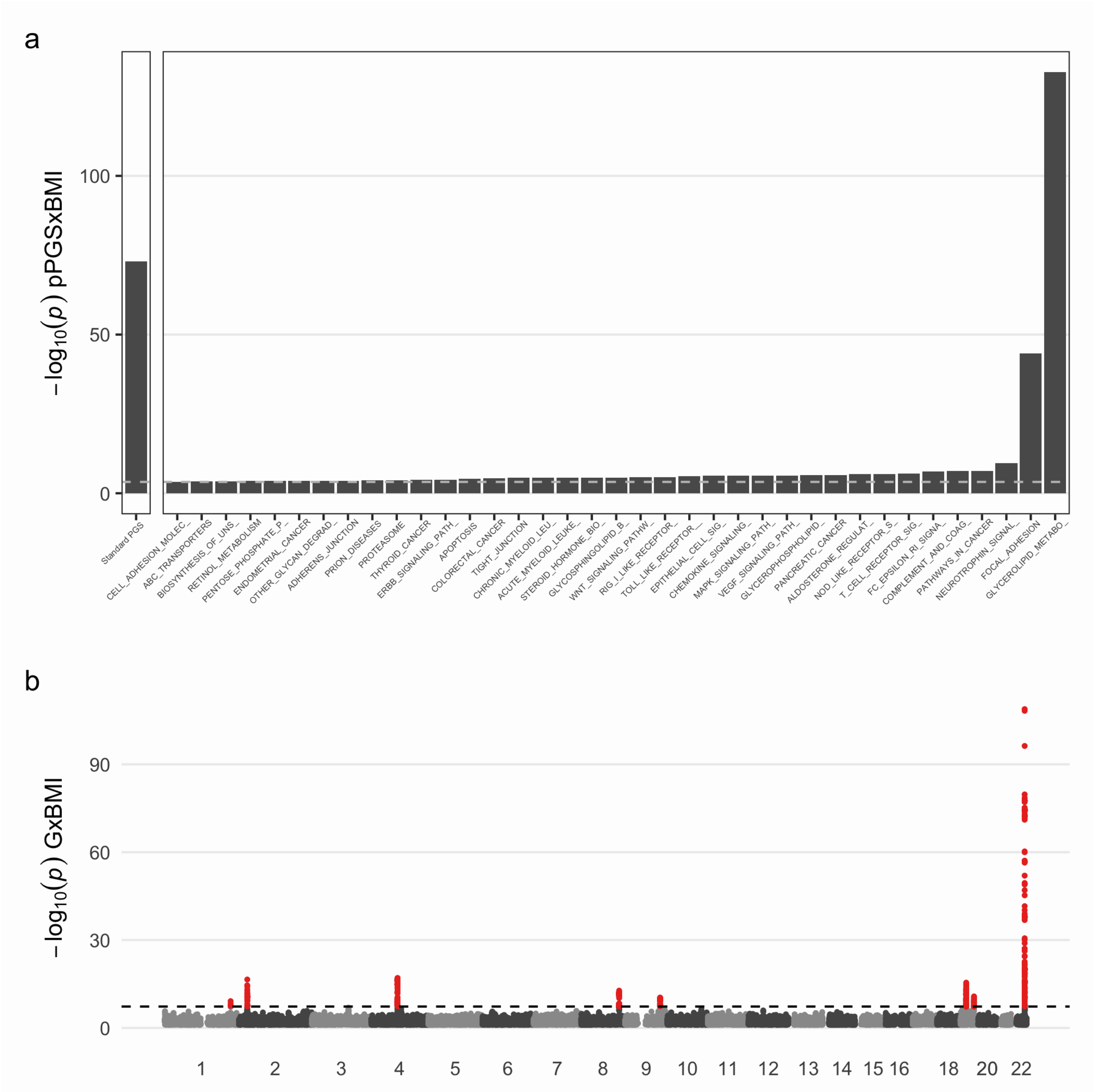

Supplementary Figure S2. GxEs shape the relationship between aspartate aminotransferase (AST) and adiposity. (a) PGSxE regression *p*-values for the gwPGS (left panel) and each significant pPGS (right panel). (b) Manhattan plot shows variant-specific *p*-values as a function of genomic position.

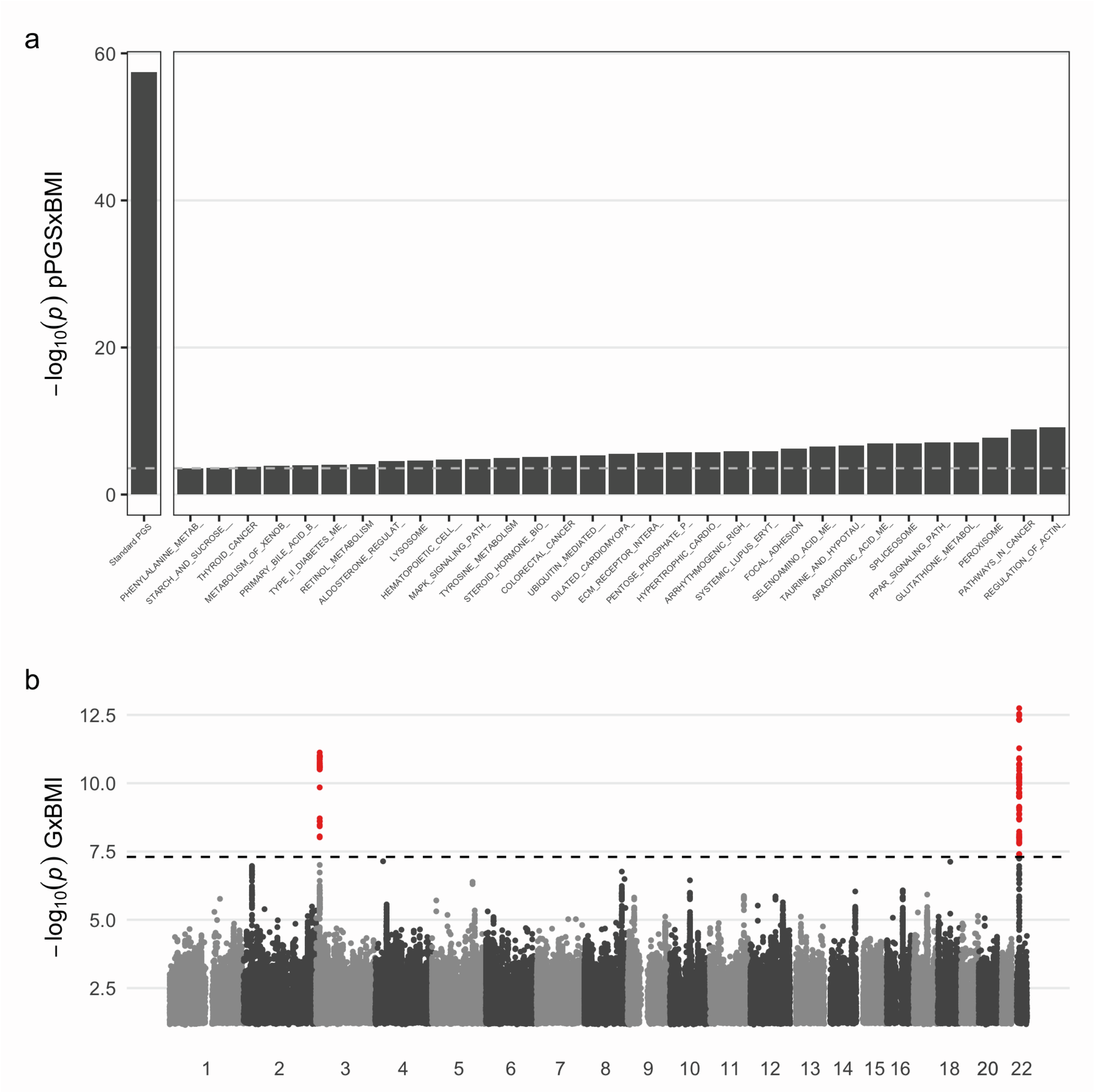

Supplementary Figure S3. GxEs shape the relationship between gamma-glutamyl transferase (GGT) and adiposity. (a) PGSxE regression *p*-values for the gwPGS (left panel) and each significant pPGS (right panel). (b) Manhattan plot shows variant-specific *p*-values as a function of genomic position.

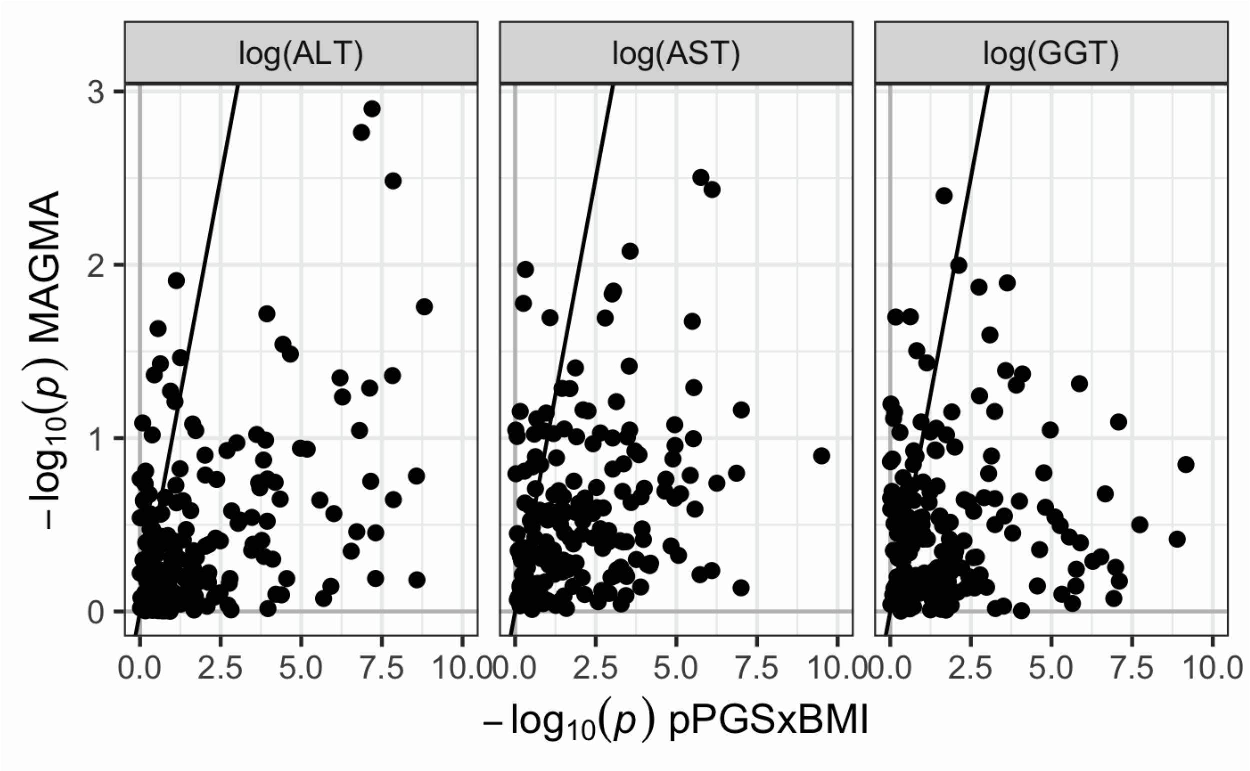

Supplementary Figure S4. Comparison of the statistical significance of pathway-level interactions tests using either pPGSxE (*x*-axis) or GWIS enrichment (based on the MAGMA tool; *y*-axis). Each point corresponds to a single KEGG legacy pathway. *x*-axis has been constrained for ALT and AST to aid visualization, which excludes results for glycerolipid metabolism and focal adhesion pathways (*p*-values reported in main text). Two points (corresponding to the "Glycerolipid Metabolism" and "Focal Adhesion" pathways) have been removed from the ALT and AST plots due to their extreme values (see Results text for their *p*-values).

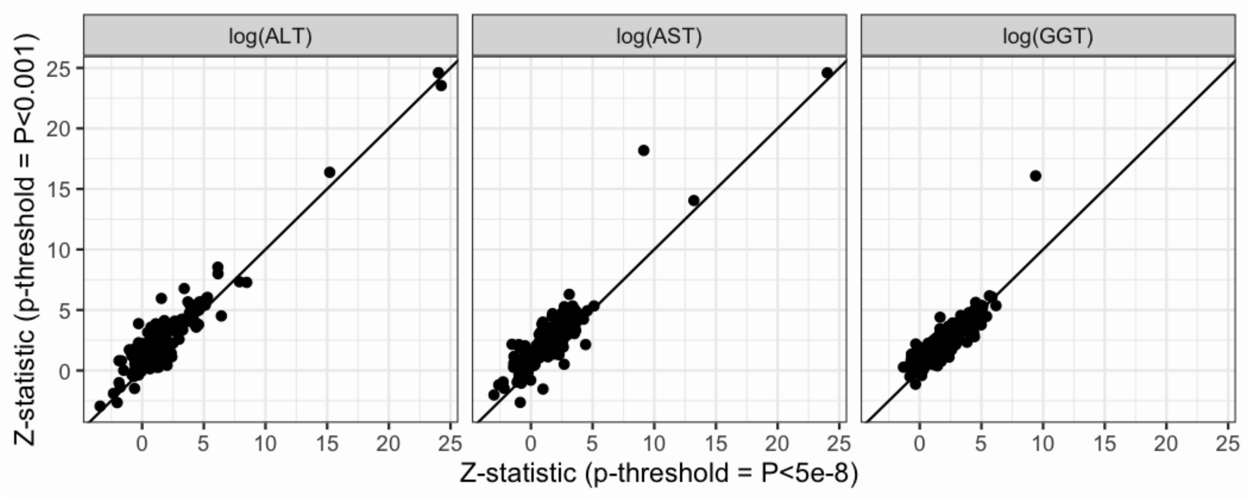

Supplementary Figure S5. Comparison of the statistical significance of pPGSxBMI tests using pPGS derived using a P&T *p*-value threshold of 0.001 (*y*-axis) versus $5x10^{-8}$ (*x*-axis). Each point corresponds to a single KEGG legacy pathway. Points could not be plotted for pathways having no annotated variants that pass $p<5x10^{-8}$.

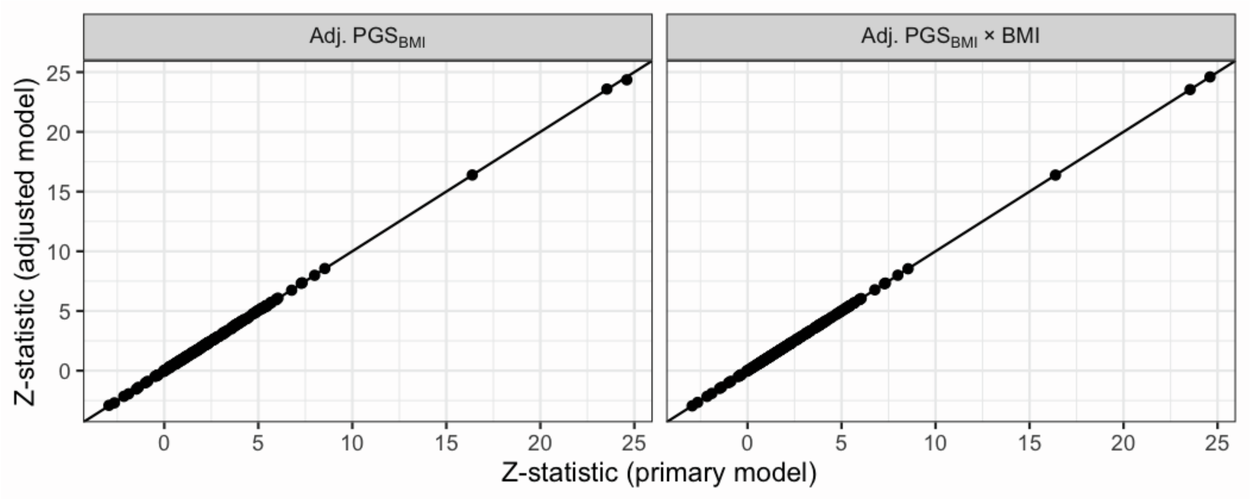

Supplementary Figure S6. Effect of PGS_{BMI} adjustment on pPGS interaction estimates. Sensitivity models adjusted for the main effect of a genome-wide PGS_{BMI} (left panel) or the main effect of PGS_{BMI} plus its interaction with physiological BMI (right panel). Interaction effect z-statistics are plotted for the primary model (*x*-axis) and adjusted model (*y*-axis).

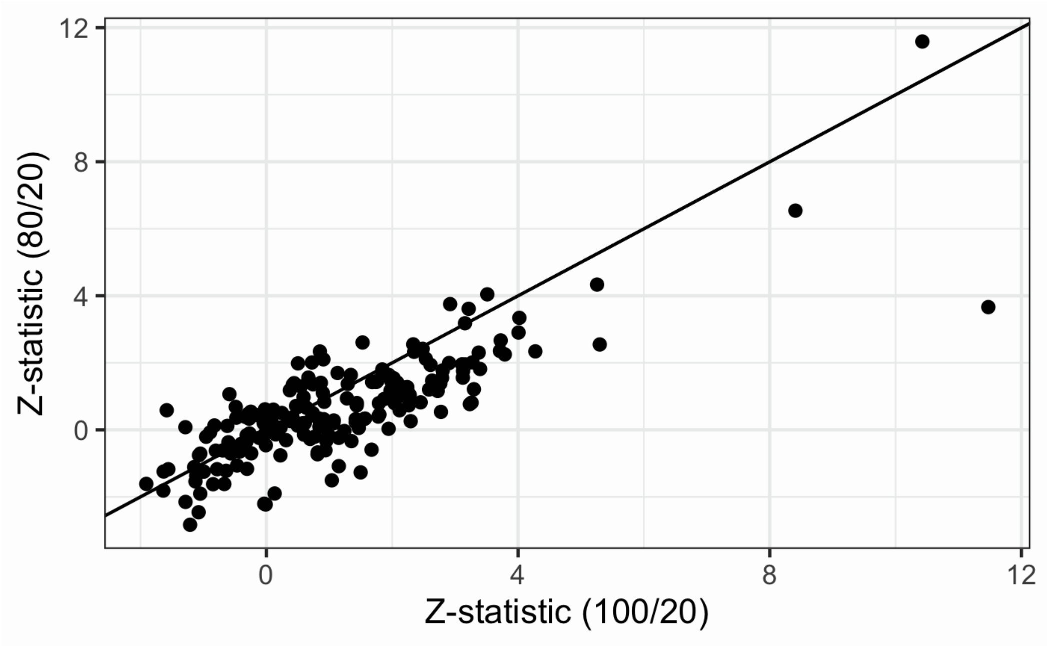

Supplementary Figure S7. Sensitivity analysis comparing primary results with those using a held-out testing set. Interaction z-statistics are compared between pPGS from the sensitivity analysis ("80/20": pPGS developed in 80% of the dataset [70% GWAS, 10% tuning] and tested in the remaining 20%) and primary analysis ("100/20": pPGS developed in 100% of the dataset and tested in the same 20%).
