# Supplementary material for: Pathway-specific polygenic scores substantially increase the discovery of gene-adiposity interactions impacting liver biomarkers
Source: HGG Adv. 2025 Sep 11;7(1):100515. doi: 10.1016/j.xhgg.2025.100515 (PMC12508838; doi:10.1016/j.xhgg.2025.100515)
Supplement: Document S2. Article plus supplemental information [file mmc3.pdf]

HGG Advances

REPORT

Pathway-specific polygenic scores substantially increase the discovery of gene-adiposity interactions impacting liver biomarkers

Kenneth E. Westerman,[1,2,3,9,*] Daniel I. Chasman,[2,4] W. James Gauderman,[5] and Arun Durvasula[6,7,8,*]

Summary

Polygenic scores (PGSs) are appealing for detecting gene-environment interactions due to the aggregation of genetic effects and reduced multiple testing burden compared to single-variant genome-wide interaction studies (GWISs). However, standard PGSs reflect many different biological mechanisms, limiting interpretation and potentially diluting pathway-specific interaction signals. Previous work has uncovered a significant genome-wide PGS×Adiposity signal impacting liver function, but there is an opportunity for additional and more interpretable discoveries. Here, we leveraged pathway-specific polygenic scores (pPGSs) to discover mechanism-specific gene-adiposity interactions. We tested for body mass index (BMI) interactions impacting three liver-related biomarkers (ALT, AST, and GGT) using (1) a standard, genome-wide PGS, (2) an array of pPGSs containing variant subsets derived from KEGG pathways, and (3) a GWIS. For ALT, we identified 49 significant pPGS×BMI interactions at a Bonferroni corrected $p < 2.7 \times 10^{-4}$, 80% of which were not explained by genes close to the 8 loci found in the associated GWIS. Across all biomarkers, we found interactions with 83 unique pPGSs. We tested alternate pathway collections (hallmark, KEGG Medicus), finding that the choice of pathway collection strongly impacts discovery. Our findings reinforced known biology (e.g., glycerolipid metabolism and hepatic lipid export affecting ALT release) and captured additional phenomena (e.g., actin cytoskeleton remodeling-associated variants alter the liver's robustness to lipid mechanical stress and thus GGT release). These results support the use of pPGSs for well powered and interpretable discovery of pPGS×E interactions with adiposity-related exposures for liver biomarkers and motivate future studies using a broader collection of exposures and outcomes.

Recent work has shown that gene-environment interactions (G×E) have a substantial impact on complex disease and trait variation.[1–6] However, discovery and interpretation of single-variant G×E via genome-wide interaction studies (GWISs) is challenging due to the large number of hypothesis tests that exacerbate the intrinsically low power of interaction tests.[7] Meanwhile, polygenic approaches, such as genome-wide polygenic score-by-E tests (gwPGS×E), may lose power by leveraging a strong and rarely satisfied assumption that main and interaction effects are proportional.[6,8,9] Recently, Durvasula and Price[6] proposed a conceptual G×E model in which environmental exposures modify genetic effects on specific pathways rather than across the entire genome, motivating G×E analyses on the pathway level as a compromise between the specificity of GWISs and the power of gwPGS×E tests.

Chasman et al.[10] provided early proof-of-concept for this approach by conducting a data-driven clustering of disease-associated genetic loci followed by cluster-specific G×E testing, focusing on cardiovascular diseases and type 2 diabetes.[11] Gauderman et al. focused on pre-established pathway annotations, showing by simulation that the use of pathway-based pPGS×E can lead to substantial increases in power over testing gwPGS×E. They also applied the approach to colorectal cancer, aggregating genome-wide significant SNPs within annotated pathways and identifying significant pPGS×E that were not detected by gwPGS×E analysis.[11] These studies underscore the potential of pathway-specific polygenic score (pPGS) approaches for increasing power to detect G×E interactions, but it remains unclear how these pPGS interactions compare to the equivalent genome-wide, variant-specific interaction study and whether the results differ substantially by choice of pathway database.

Leveraging the annotation-based pathway approach, we sought to explore genetic modification of the relationship between adiposity (as measured by body mass index [BMI]) and liver stress biomarkers. Our prior work demonstrated the presence of a polygenic signature modifying the relationship between BMI and cardiometabolic risk factors.[12] There was a particularly strong signature for

[1]Clinical and Translational Epidemiology Unit, Massachusetts General Hospital, Boston, MA, USA; [2]Department of Medicine, Harvard Medical School, Boston, MA, USA; [3]Programs in Metabolism and Medical and Population Genetics, Broad Institute of MIT and Harvard, Cambridge, MA, USA; [4]Division of Preventive Medicine, Brigham and Women's Hospital, Boston, MA, USA; [5]Division of Biostatistics, Department of Population and Public Health Sciences, Keck School of Medicine, University of Southern California, Los Angeles, CA, USA; [6]Division of Epidemiology, Department of Population and Public Health Sciences, Keck School of Medicine, University of Southern California, Los Angeles, CA, USA; [7]Center for Genetic Epidemiology, Department of Population and Public Health Sciences, Keck School of Medicine, University of Southern California, Los Angeles, CA, USA; [8]Department of Quantitative and Computational Biology, University of Southern California, Los Angeles, CA, USA
[9]Lead contact
*Correspondence: kewesterman@mgb.org (K.E.W.), arun.durvasula@med.usc.edu (A.D.)
https://doi.org/10.1016/j.xhgg.2025.100515.
© 2025 The Author(s). Published by Elsevier Inc. on behalf of American Society of Human Genetics.
This is an open access article under the CC BY license (http://creativecommons.org/licenses/by/4.0/).

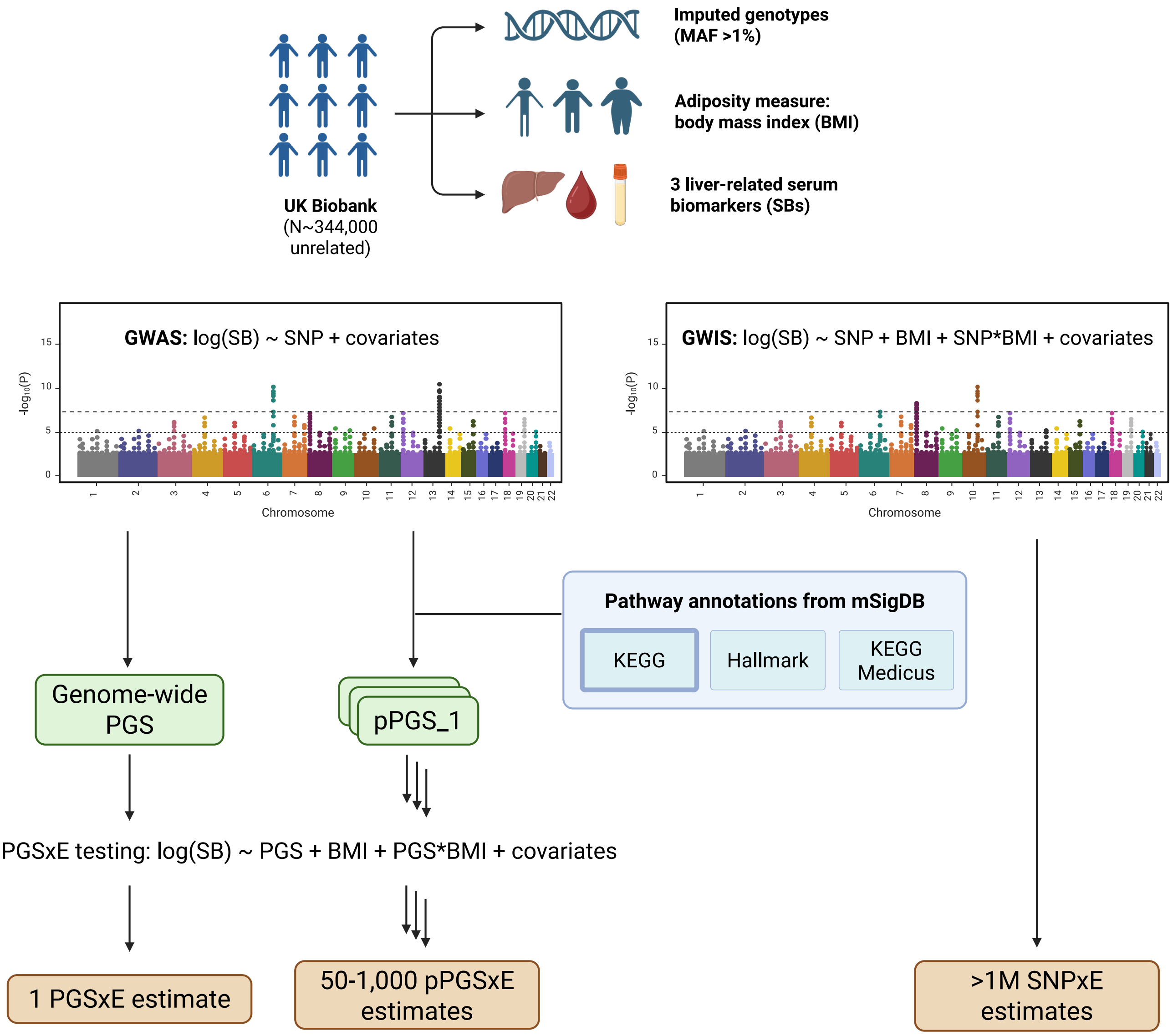

Figure 1. Pipeline for multi-scale gene-by-body mass index interaction (G×BMI) testing carried out in this study

liver-related biomarkers, which are indicative of multiple types of liver damage (see supplemental methods). We showed preliminary evidence that interactions with BMI reflected a mechanism-specific subset of the overall genetic architecture of these biomarkers. Here, we explore this biological question further as an applied setting to test the incorporation of existing biological annotations and pPGSs into an interaction testing framework.

Our analysis pipeline is summarized in Figure 1. We analyzed individual-level data for 344,000 unrelated participants of European ancestry from the UK Biobank (UKB). We performed a standard GWAS for each of three log-transformed liver-related biomarkers (alanine aminotransferase [ALT], aspartate aminotransferase [AST], and gamma-glutamyl transferase [GGT]) in the entire UKB dataset. We used these summary statistics to create a genome-wide PGS (gwPGS) as well as a series of pPGSs based on KEGG (Kyoto Encyclopedia of Genes and Genomes) pathway annotations. We then tested each PGS for interaction with BMI. For comparison to the PGS-based interaction tests, we performed a GWIS for each biomarker with BMI as the exposure.

We first tested for the interaction between genome-wide PGS and BMI in a regression including a main effect for the gwPGS and adjusting for age, age^2, sex, age×sex, 10 genomic principal components (gPCs), and 10 BMI×gPC product terms. We used a pruning and thresholding (P&T) approach to train the gwPGSs, retaining between 4612 and 6164 SNPs at a p value threshold of 0.001 (across the three biomarkers). We identified strong gwPGS×E interactions for all three liver biomarkers. For ALT, AST, and GGT, these interaction effect sizes were 0.035 $SD_{log(ALT)}/SD_{PGS}/SD_{BMI}$ (SD, standard deviation; $p = 1.7 \times 10^{-133}$), 0.028 ($p = 7.8 \times 10^{-74}$), and 0.022 ($p = 3.7 \times 10^{-58}$), respectively. These results are concordant with our recent analyses of PGS×BMI for liver

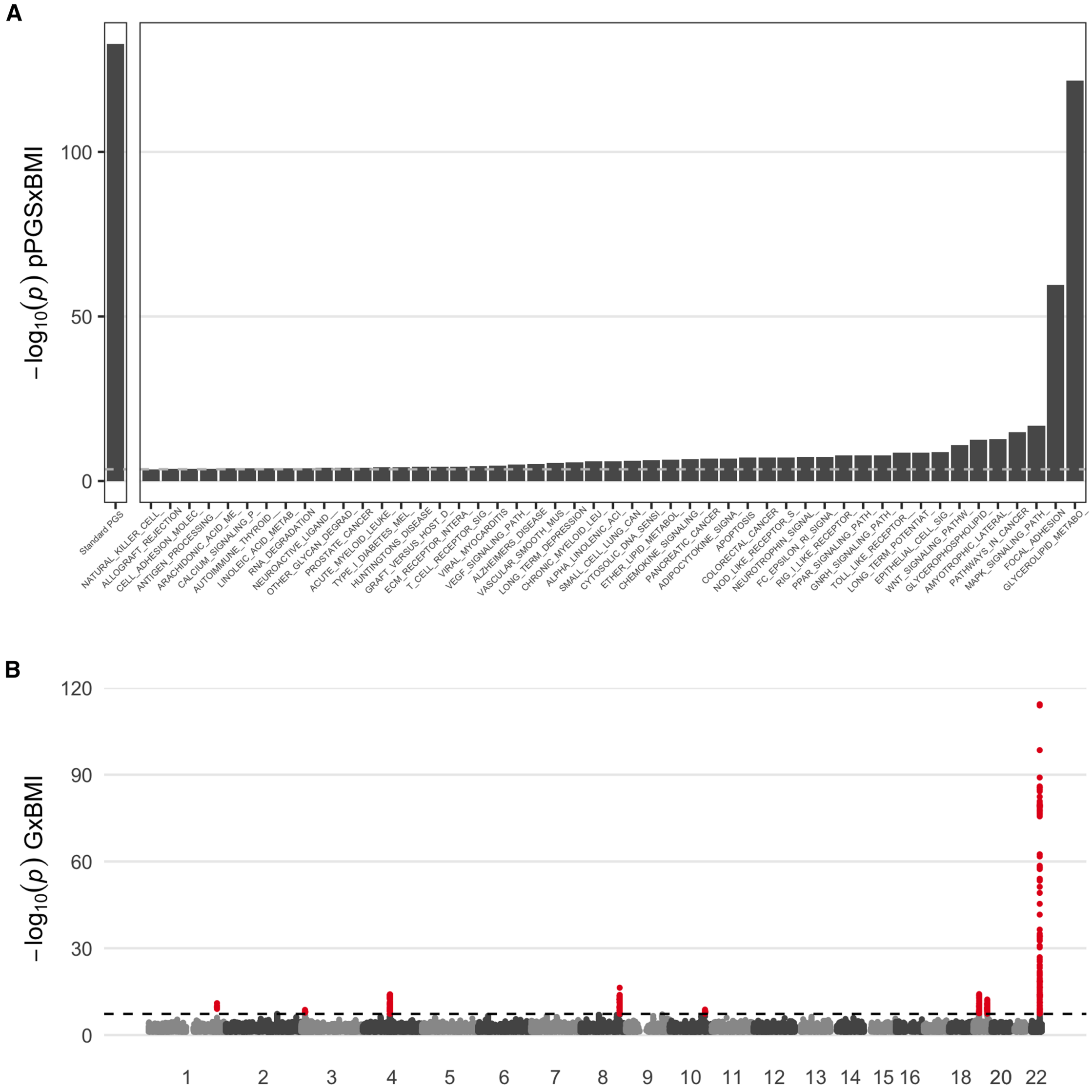

Figure 2. G×Es shape the relationship between adiposity and alanine aminotransferase (ALT)
(A) PGS×E regression p values for the gwPGS (left) and each significant pPGS (right). x axis pathway labels are truncated for the purpose of visualization.
(B) Manhattan plot shows SNP-specific p values as a function of genomic position. Results for AST and GGT are provided in Figures S2 and S3. Numeric results for all biomarkers are provided in Tables S1 and S2.

biomarkers,[12] and all represent patterns in which higher PGS values magnify the positive BMI-biomarker relationship. We note that there is no expected bias due to training a standard PGS and testing it for interaction in the same dataset, as demonstrated by Gauderman and colleagues.[11]

Next, we investigated the discovery enabled by pathway-level testing. We assigned SNPs to genes based on physical distance to the closest annotated gene (−2 kb to +1 kb from the gene transcription start site and transcription end site, respectively) and assigned genes to pathways using gene sets from the mSigDB database, focusing on the KEGG gene-to-pathway mappings as the primary pathway group of interest (Figure S1). We calculated the pPGS for each pathway using a p-value-based P&T approach as implemented in the PRSet program,[13] using a p value threshold of 0.001. We tested for interactions between pPGS and BMI using regression models analogous to those used for the standard gwPGS above, assigning significance based on a Bonferroni correction for the 186 total pathways tested. We identified a substantial number of significant pPGS×E for all three biomarkers (Figure 2B for ALT; full set of results in Table S1). First, we found 49 significant pPGS×E interactions for ALT. Though no single pPGS reached the same degree of significance as the gwPGS, interactions were highly significant for multiple pathways, including glycerolipid metabolism ($p = 2.0 \times 10^{-122}$) and focal adhesion ($p = 2.9 \times 10^{-60}$) (Figure 2A). The glycerolipid metabolism association is concordant with previous work showing a

Table 1. **Overlap between significant findings from the single-variant GWIS and pPGS approaches**

Biomarker	# GWIS loci	# pPGS×BMI	# "Unexplained" pPGS×BMI pathways	Fraction "unexplained" pPGS×BMI pathways
ALT	8	49	39	80%
AST	8	37	34	92%
GGT	2	31	22	71%

relationship between glycerolipid metabolism and obesity[14] as well as obesity and liver biomarkers.[15] Results for AST were similar, with the same top two pathways and 37 significant pathways in total (Figure S2). Notably, for AST, the same glycerolipid metabolism pathway reached a greater degree of significance than the gwPGS ($p = 2.1 \times 10^{-133}$), highlighting the value of the pPGS in improving power by prioritizing relevant genomic regions. GGT analyses resulted in 31 significant pathways, with a notably different pattern of significance across pathways compared to ALT and AST, suggesting a different architecture of G×Es (Figure S3). Here, the top pathways included regulation of actin cytoskeleton, pathways in cancer, peroxisome, and glutathione metabolism. The collection of all pPGS interactions added substantial signal in aggregate: models that included all pPGS×BMI interactions effects fit the data significantly better than models with only gwPGS×BMI interaction effects (ALT $p = 5 \times 10^{-66}$, AST $p = 4 \times 10^{-99}$, GGT $p = 1 \times 10^{-7}$; see supplemental methods for description of likelihood ratio tests). Taken together, these results show that pPGS×E analysis can break down genome-wide gwPGS×E signals into biologically interpretable results.

Next, we performed a single-variant GWIS for each biomarker using analogous regression models to that for the gwPGS and pPGS, additionally using robust standard errors and applying a genome-wide significance threshold of 5×10^{-8}. We found 8 genome-wide significant loci for each of ALT and AST (7 of these were overlapping) and 2 for GGT (Figures 1B, S2, and S3; Table S2) after a simple distance-based pruning (±500 kb from lead variants). The strongest locus for both ALT and AST mapped to the *PNPLA3* gene on chromosome 22, a well-known locus affecting liver disease and metabolic traits,[16] whose strong main effect on ALT and AST has previously been shown to be modified by BMI in UKB.[12] All 8 genome-wide significant GWIS loci also achieved significance in the associated GWAS for liver biomarkers, in analysis examining solely main effects.

Next, we asked how many of the pPGS×E results overlapped with biological pathways that could have been discovered via the GWIS approach. We annotated pPGS×E tests as "explained" if there was at least one GWIS lead variant near any gene in that pathway set (within 100 kb of the TSS based on Ensembl GRCh37 v75). For ALT, AST, and GGT, we found that 39 (80%), 34 (92%), and 22 (71%) pathway interactions were unexplained by significant GWIS loci (Table 1), respectively. These findings indicate that the majority of pPGS×E interactions point to pathways that would not have been found using a single-variant approach. When testing for enrichment of GWIS signal using the MAGMA tool and the same set of variant-gene-pathway annotations, we found that results were significantly correlated with those from the pPGS×E approach ($r_{Spearman} = 0.25$ for $-\log_{10}(p)$ values, $p = 2.8 \times 10^{-9}$) but systematically less significant (Figure S4). In addition, all pathways highlighted by this GWIS enrichment approach were discovered by the pPGS×E approach. The two strongest pathways from the pPGS×E approach for ALT and AST (glycerolipid metabolism and focal adhesion) showed minimal signal in the enrichment-based approach ($p > 0.05$ for both). Taken together, these results highlight the increased power of pPGS×E to discover G×E interactions over GWIS.

To understand the impact of the choice of pathway database (i.e., gene-to-pathway mapping) on our results, we reran the same analysis pipeline using two additional pathway collections from mSigDB: hallmark pathways and KEGG Medicus (see Figure S1 for metadata describing these collections). Over all biomarkers, these collections resulted in 83 (KEGG), 36 (hallmark), and 67 (KEGG Medicus) significant pPGS×BMI interactions. The comparison of KEGG Medicus, an expansion of KEGG to include disease- and drug-related annotations, to the primary KEGG "legacy" collection indicates the importance of pathway collection choice: fewer significant pathways were uncovered despite a much larger number of available pathways (658 for Medicus versus 186 for legacy) due to the specific biological pathways represented and the differential multiple testing burden.

We used a series of secondary analyses to address potential concerns. First, we chose a single P&T p value threshold of 0.001 based on optimized main effect PGS thresholds from a prior UKB analysis.[12] We confirmed that this choice did not substantially impact the pPGS×E results: interaction effects based on pPGS using a P&T threshold of 5×10^{-8} showed minimal difference in significance (Figure S5). Second, we calculated an adjusted (or "effective") number of pathways discovered to account for the fact that some pathways are correlated (see supplemental methods for details on the PCA-based method). This resulted in a reduction of significant KEGG pathways from 49 to 9.6 effective pathways for ALT, 21 to 11.1 for AST, and 10 to 2.8 for GGT. Third, we verified that the uncovered interactions were not due solely to the pPGS tracking with the genetics of BMI: interaction estimates changed minimally when adjusting models for a genome-wide PGS for BMI (PGS_{BMI}) or,

additionally, a $PGS_{BMI}\times BMI$ product term (Figure S6). Finally, we confirmed that developing pPGS and testing for interactions in the same sample set does not induce bias, which was previously studied using simulations.[11] We repeated our pPGS×E interaction tests in a 20% subset of the UKB and found similar results when using pPGS developed in the rest of the dataset as opposed to the full dataset (Figure S7; supplemental methods).

We leveraged pathway annotations and the UK Biobank with complex trait, exposure, and genetic data to identify gene-environment interactions acting on specific pathways. We found 83 significant KEGG pathway pPGS×BMI interactions across three liver biomarkers, pointing to specific biological processes modifying the relationship between adiposity and liver health.

The highly significant glycerolipid pathway finding for ALT and AST captures an interaction that we and others have described: genetic effects on this pathway's function alter the liver's ability to relieve adiposity-associated lipid buildup.[17] The top hit for GGT was regulation of actin cytoskeleton; this may point to the importance of cytoskeletal and mechanical integrity for maintaining proper bile flow, whose deterioration impacts GGT more than ALT or AST.[18] Obesity-associated lipid buildup can increase mechanical stress in hepatocytes[19] and disrupt the bile canaliculus network,[20] with potential modification of this effect by genetic effects on cytoskeletal integrity. While the preceding pathways have been studied in the context of liver health, additional highly significant pathways from the pPGS interaction approach may point to additional mechanisms of obesity-related liver pathogenesis. For example, the disease-associated amyotrophic lateral sclerosis (ALS) pathway was one of the strongest for ALT. While ALS is a motor neuron disease not classically associated with liver dysfunction, patients are highly enriched for hepatic steatosis, possibly via mitochondrial and endoplasmic reticulum stress pathways,[21] suggesting directions for further study.

Our study represents an advance over previous studies investigating pathway-specific polygenic scores for interaction. Our approach based on *a priori* pathway annotations increases the interpretability of our pPGS×E results compared to data-driven approaches. Leveraging these strengths, we show that pPGS×E testing enables increased discovery of gene-environment interactions and produces interactions that (1) are mostly unexplained by the associated variant-specific GWIS and (2) can be stronger than the associated gwPGS×E test. Furthermore, we show that the choice of pathway annotation (variant to gene to pathway) has a substantial impact on pPGS×E results and more comprehensive exploration of additional pathway collections is likely to further increase the number of interactions uncovered.

Our study has several limitations. First, we used a simple distance-based method for variant-to-gene mapping and a limited set of pathway annotations. Future work could apply more sophisticated and wide-ranging methods making use of functional genomic data to assign SNPs to genes and ultimately pathways. Second, we study a limited subset of biomarkers and only one exposure. Our methodological conclusions may not hold universally across all biomarkers and exposures. Third, we did not account for correlated pathway annotations, which makes our stringent Bonferroni correction for the total number of pathways conservative. Future work could model the correlated tests to increase statistical power. Despite these limitations, our work highlights pathway-specific PGS×E testing as a powerful way to discover G×Es.

Acknowledgments

K.E.W. was supported by National Institutes of Health K01DK133637. A.D. was supported by NIH R35GM160467.

Declaration of interests

The authors declare no competing interests.

Supplemental information

Supplemental information can be found online at https://doi.org/10.1016/j.xhgg.2025.100515.

Received: June 8, 2025
Accepted: September 8, 2025

References

1. Wang, H., Zhang, F., Zeng, J., Wu, Y., Kemper, K.E., Xue, A., Zhang, M., Powell, J.E., Goddard, M.E., Wray, N.R., et al. (2019). Genotype-by-environment interactions inferred from genetic effects on phenotypic variability in the UK Biobank. Sci. Adv. *5*, eaaw3538.
2. Westerman, K.E., Majarian, T.D., Giulianini, F., Jang, D.K., Miao, J., Florez, J.C., Chen, H., Chasman, D.I., Udler, M.S., Manning, A.K., and Cole, J.B. (2022). Variance-quantitative trait loci enable systematic discovery of gene-environment interactions for cardiometabolic serum biomarkers. Nat. Commun. *13*, 3993.
3. Zhu, C., Ming, M.J., Cole, J.M., Edge, M.D., Kirkpatrick, M., and Harpak, A. (2023). Amplification is the primary mode of gene-by-sex interaction in complex human traits. Cell Genom. *3*, 100297.
4. Pazokitoroudi, A., Liu, Z., Dahl, A., Zaitlen, N., Rosset, S., and Sankararaman, S. (2024). A scalable and robust variance components method reveals insights into the architecture of gene-environment interactions underlying complex traits. Am. J. Hum. Genet. *111*, 1462–1480.
5. Herrera-Luis, E., Benke, K., Volk, H., Ladd-Acosta, C., and Wojcik, G.L. (2024). Gene-environment interactions in human health. Nat. Rev. Genet. *25*, 768–784.
6. Durvasula, A., and Price, A.L. (2025). Distinct explanations underlie gene-environment interactions in the UK Biobank. Am. J. Hum. Genet. *112*, 644–658.
7. Gauderman, W.J., Mukherjee, B., Aschard, H., Hsu, L., Lewinger, J.P., Patel, C.J., Witte, J.S., Amos, C., Tai, C.G., Conti,

D., et al. (2017). Update on the State of the Science for Analytical Methods for Gene-Environment Interactions. Am. J. Epidemiol. *186*, 762–770.
8. Zhai, S., Zhang, H., Mehrotra, D.V., and Shen, J. (2022). Pharmacogenomics polygenic risk score for drug response prediction using PRS-PGx methods. Nat. Commun. *13*, 5278.
9. Aschard, H. (2016). A perspective on interaction effects in genetic association studies. Genet. Epidemiol. *40*, 678–688.
10. Chasman, D.I., Giulianini, F., Demler, O.V., and Udler, M.S. (2020). Pleiotropy-based decomposition of genetic risk scores: Association and interaction analysis for type 2 diabetes and CAD. Am. J. Hum. Genet. *106*, 646–658.
11. Gauderman, W.J., Fu, Y., Queme, B., Kawaguchi, E., Wang, Y., Morrison, J., Brenner, H., Chan, A., Gruber, S.B., Keku, T., and Li, L. (2025). Pathway polygenic risk scores (pPRS) for the analysis of gene-environment interaction. PLoS Genet. *21*, e1011543.
12. Westerman, K.E., Gervis, J.E., O'Connor, L.J., Udler, M.S., and Manning, A.K. (2025). Polygenic scores capture genetic modification of the adiposity-cardiometabolic risk factor relationship. Preprint at medRxiv. https://doi.org/10.1101/2025.04.09.25324066.
13. Choi, S.W., García-González, J., Ruan, Y., Wu, H.M., Porras, C., Johnson, J., Bipolar Disorder Working group of the Psychiatric Genomics Consortium, Hoggart, C.J., and O'Reilly, P.F. (2023). PRSet: Pathway-based polygenic risk score analyses and software. PLoS Genet. *19*, e1010624.
14. Prentki, M., and Madiraju, S.R.M. (2012). Glycerolipid/free fatty acid cycle and islet β-cell function in health, obesity and diabetes. Mol. Cell. Endocrinol. *353*, 88–100.
15. Jalili, V., Poorahmadi, Z., Hasanpour Ardekanizadeh, N., Gholamalizadeh, M., Ajami, M., Houshiarrad, A., Hajipour, A., Shafie, F., Alizadeh, A., Mokhtari, Z., et al. (2022). The association between obesity with serum levels of liver enzymes, alanine aminotransferase, aspartate aminotransferase, alkaline phosphatase and gamma-glutamyl transferase in adult women. Endocrinol. Diabetes Metab. *5*, e367.
16. Souza, M., Al-Sharif, L., Diaz, I., Mantovani, A., and Villela-Nogueira, C.A. (2025). Global epidemiology and implications of PNPLA3 I148M variant in metabolic dysfunction-associated steatotic liver disease: A systematic review and meta-analysis. J. Clin. Exp. Hepatol. *15*, 102495.
17. Gao, C., Marcketta, A., Backman, J.D., O'Dushlaine, C., Staples, J., Ferreira, M.A.R., Lotta, L.A., Overton, J.D., Reid, J. G., Mirshahi, T., et al. (2021). Genome-wide association analysis of serum alanine and aspartate aminotransferase, and the modifying effects of BMI in 388k European individuals. Genet. Epidemiol. *45*, 664–681.
18. Thakur, S., Kumar, V., Das, R., Sharma, V., and Mehta, D.K. (2024). Biomarkers of hepatic toxicity: An overview. Curr. Ther. Res. Clin. Exp. *100*, 100737.
19. Loneker, A.E., Alisafaei, F., Kant, A., Li, D., Janmey, P.A., Shenoy, V.B., and Wells, R.G. (2023). Lipid droplets are intracellular mechanical stressors that impair hepatocyte function. Proc. Natl. Acad. Sci. USA *120*, e2216811120.
20. Shek, D., Chen, D., Read, S.A., and Ahlenstiel, G. (2021). Examining the gut-liver axis in liver cancer using organoid models. Cancer Lett. *510*, 48–58.
21. Parekh, B. (2015). A (a)LS: Ammonia-induced amyotrophic lateral sclerosis. F1000Res. *4*, 119.

HGGA, Volume 7

Supplemental information

Pathway-specific polygenic scores substantially increase the discovery of gene-adiposity interactions impacting liver biomarkers

Kenneth E. Westerman, Daniel I. Chasman, W. James Gauderman, and Arun Durvasula

Supplementary Methods

UK Biobank data

We used data from the large, prospective UK Biobank cohort in all analyses[1]. This research was conducted using the UK Biobank resource under application no. 277892 and Not Human Subjects Research determination NHSR-4298 at the Broad Institute of MIT and Harvard. Genotyping, imputation, and initial quality control on the genetic dataset have been described previously[2]. Work was conducted on genetic data release version 3, with imputation to both Haplotype Reference Consortium[3] and 1000 Genomes Project (1KGP)[4]. Ultimately, analysis was performed on a set of unrelated individuals, defined as the set of individuals whose genomes were included in centrally performed genetic principal components analysis. As described in prior related work[5] , we excluded individuals that had withdrawn consent by the time of analysis excluded as well as those with diabetes, coronary heart disease, cirrhosis, end-stage renal disease, cancer diagnosis within one year prior to their assessment center visit, or who were pregnant within one year of the assessment center visit.

Body mass index (BMI; kg/m^2) was collected from assessment center anthropometric measurements. Serum biomarker values were measured in blood samples collected at the baseline visit (details available at: https://biobank.ctsu.ox.ac.uk/crystal/crystal/docs/serum_biochemistry.pdf).

Liver biomarkers

We focused our analyses on three biomarkers for liver function: alanine aminotransferase (ALT), aspartate aminotransferase (AST), and gamma-glutamyl transferase (GGT). These biomarkers are used to diagnose liver diseases, including non-alcoholic fatty liver disease[6]. ALT is the most liver-specific of these biomarkers, with elevation indicating hepatocellular injury[7]. AST is also elevated with hepatocellular injury, but is more closely tied to alcohol-related steatohepatitis and can also suggest other sources of physiological stress, such as myopathy[7]. GGT elevation, in contrast, is often indicative of a cholestatic pattern involving bile duct obstruction[7]. Due to their skewed distribution, we log-transformed each biomarker prior to analysis. We avoid issues of apparent interactions caused by scale effects for binary outcomes by focusing on biomarker levels rather than disease status[8].

Genome-wide studies

A genome-wide association study (GWAS) and genome-wide interaction study (GWIS) was performed for each log-transformed biomarker. The GWAS used a basic linear model corrected for covariates:

$$Y = G + C,$$

We adjusted for the following covariates: age, age^2, sex, and ten genetic PCs. We used model-based standard errors and included only variants with minor allele frequency >1% and imputation quality INFO score >0.5 (approximately 9,891,000 variants in total). The GWIS model added an environmental exposure and its product term with G:

$$Y = G + E + G \times E + C,$$

In the GWIS, we additionally adjusted for an E×gPC product term for each gPC[9] and used robust standard errors. All genome-wide studies were conducted using GEM v1.5.2[10].

Pathway annotations and polygenic score generation

We generated PGS from GWAS summary statistics using the PRSet program[11], which builds on PRSice-2[12]. PRSet both computes pathway-specific PGS weights (using the P&T approach along with an LD reference panel) and calculates scores for the input UKB dataset as a linear combination of genotypes based on those weights. As input parameters governing the behavior of the P&T algorithm, we used p-value thresholds of both 0.001 and $5\text{x}10^{-8}$, a clumping radius of 1MB, an r^2 threshold of 0.1, and an LD reference panel consisting of a random 20,000 individuals from the UKB. In all analyses, we considered only autosomal variants and removed ambiguous variants (A/T or C/G) during PGS development.

To generate pPGS, we additionally obtained pathway annotations from mSigDB[13]. We used three sets of pathway collections: 1) KEGG[14], 2) Hallmark[13], and 3) KEGG Medicus[14]. PRSet assigns variants to pathways via physical proximity to constituent genes and subsequently conducts a separate P&T procedure for each pathway. For this variant to gene mapping, we included variants within a boundary of 2kb upstream (5') and 1kb downstream (3') of the gene transcription start site and end site, respectively. By default, PRSet also generates a gwPGS using the same P&T procedure but including all available variants, resulting in gwPGS containing 4,612, 4,894, and 6,164 variants for ALT, AST, and GGT, respectively.

Polygenic score by environment testing

We tested for interactions using linear regression of the form:

$$Y = PGS + E + PGS \times E + C,$$

where Y is the trait value, PGS is the polygenic score for the trait, E is the environment variable, and C is a set of covariates. For pathway PGS, we used the following linear regression for a single pathway:

$$Y = pPGS_i + E + pPGS_i \times E + C,$$

Where $i \in P$ indexes the pathway in the set of pathways P (see Pathway annotations and polygenic score generation). For all analyses, we correct for the same covariates as in the GWIS: age, age^2, sex, ten genetic PCs, and an E×gPC product term for each gPC[9]. We used the p-value associated with the interaction term to assess significance. To account for multiple testing in the pathway specific PGS, we used a Bonferroni corrected threshold of 0.05 divided by the number of pathways tested. All individual-level data preprocessing and regression analysis after PGS generation was performed using R v4.1 and 4.2[15] except where otherwise noted.

We used likelihood ratio tests to understand whether the cumulative contribution of many pPGS×BMI interactions explained significantly more variance than the gwPGS×BMI interaction alone. The restricted model included all covariates from the basic pPGS×BMI interaction tests (see above) as well as a main effect for the gwPGS, main effects for each pPGS, and an interaction term for the gwPGS. The full model added interaction terms for each of the pPGS. We tested for significance of the additional variance explained by the full model using the *lmtest::lrtest()* function.

To enable us to a report a number of discoveries that accounted for the substantial correlation between pPGS, we calculated an "effective" number of pathways discovered for each biomarker

using a previously described method[16]. Briefly, we subsetted a rectangular matrix of individual-level pPGS values to include only those that were significant for the biomarker of interest, then performed principal components analysis (*prcomp* function with standardized variables). The number of effective biomarkers was then calculated from the principal component variances λ (equal to the eigenvalues of the biomarker covariance matrix) as $N_{BM,eff} = \frac{\left(\sum_{k=1}^{p} \lambda_k\right)^2}{\sum_{k=1}^{p} \lambda_k^2}$.

Sensitivity analyses

To understand whether the pPGS interactions were due to their prediction of BMI (rather than liver biomarkers), we also generated a genome-wide PGS for BMI (as the outcome phenotype). We followed the same procedure as used for each liver biomarker, selecting only the whole-genome score for downstream sensitivity analysis.

To evaluate the impact of our "in sample" training and testing procedure, we performed an additional analysis mirroring the original, but using separate training (70%), tuning (10%), and testing (20%) data subsets. Unlike the original analysis, this enabled the selection of an optimal *p*-value threshold for each pathway based on prediction performance in the tuning set (thresholds in $\{5\times10^{-8}, 5\times10^{-7}, 5\times10^{-6}, \ldots, 0.05\}$). These optimized pPGS were then tested for interaction with BMI in the held-out testing set. Importantly, the results from this analysis ("80/20") were compared with those using pPGS from the primary analysis but tested in the same 20% of participants ("100/20"). We note that in this comparison, beyond any influence of sample overlap, the 100/20 analysis benefits slightly from having conducted the GWAS in a larger sample.

Supplementary References

1. Sudlow, C. *et al.* UK biobank: an open access resource for identifying the causes of a wide range of complex diseases of middle and old age. *PLoS Med.* **12**, e1001779 (2015).
2. Bycroft, C. *et al.* The UK Biobank resource with deep phenotyping and genomic data. *Nature* **562**, 203–209 (2018).
3. McCarthy, S. *et al.* A reference panel of 64,976 haplotypes for genotype imputation. *Nat. Genet.* **48**, 1279–1283 (2016).
4. Byrska-Bishop, M. *et al.* High-coverage whole-genome sequencing of the expanded 1000 Genomes Project cohort including 602 trios. *Cell* **185**, 3426-3440.e19 (2022).
5. Westerman, K. E., Gervis, J. E., O'Connor, L. J., Udler, M. S. & Manning, A. K. Polygenic scores capture genetic modification of the adiposity-cardiometabolic risk factor relationship. *medRxiv* 2025.04.09.25324066 (2025) doi:10.1101/2025.04.09.25324066.
6. Sanyal, A. J. *et al.* Diagnostic performance of circulating biomarkers for non-alcoholic steatohepatitis. *Nat. Med.* **29**, 2656–2664 (2023).
7. Lala, V., Zubair, M. & Minter, D. A. Liver function tests. in *StatPearls* (StatPearls Publishing, Treasure Island (FL), 2025).
8. Durvasula, A. & Price, A. L. Distinct explanations underlie gene-environment interactions in the UK Biobank. *Am. J. Hum. Genet.* **112**, 644–658 (2025).
9. Keller, M. C. Gene × environment interaction studies have not properly controlled for potential confounders: the problem and the (simple) solution. *Biol. Psychiatry* **75**, 18–24 (2014).

10. Westerman, K. E. *et al.* GEM: scalable and flexible gene-environment interaction analysis in millions of samples. *Bioinformatics* **37**, 3514–3520 (2021).
11. Choi, S. W. *et al.* PRSet: Pathway-based polygenic risk score analyses and software. *PLoS Genet.* **19**, e1010624 (2023).
12. Choi, S. W. & O'Reilly, P. F. PRSice-2: Polygenic Risk Score software for biobank-scale data. *Gigascience* **8**, giz082 (2019).
13. Liberzon, A. *et al.* The Molecular Signatures Database (MSigDB) hallmark gene set collection. *Cell Syst.* **1**, 417–425 (2015).
14. Kanehisa, M., Furumichi, M., Tanabe, M., Sato, Y. & Morishima, K. KEGG: new perspectives on genomes, pathways, diseases and drugs. *Nucleic Acids Res.* **45**, D353–D361 (2017).
15. R Core Team. R: A language and environment for statistical computing. *R Foundation for Statistical Computing, Vienna, Austria. URL https://www.r-project.org/* (2022).
16. Wang, H. *et al.* Genotype-by-environment interactions inferred from genetic effects on phenotypic variability in the UK Biobank. *Sci Adv* **5**, eaaw3538 (2019).

Supplementary Figures

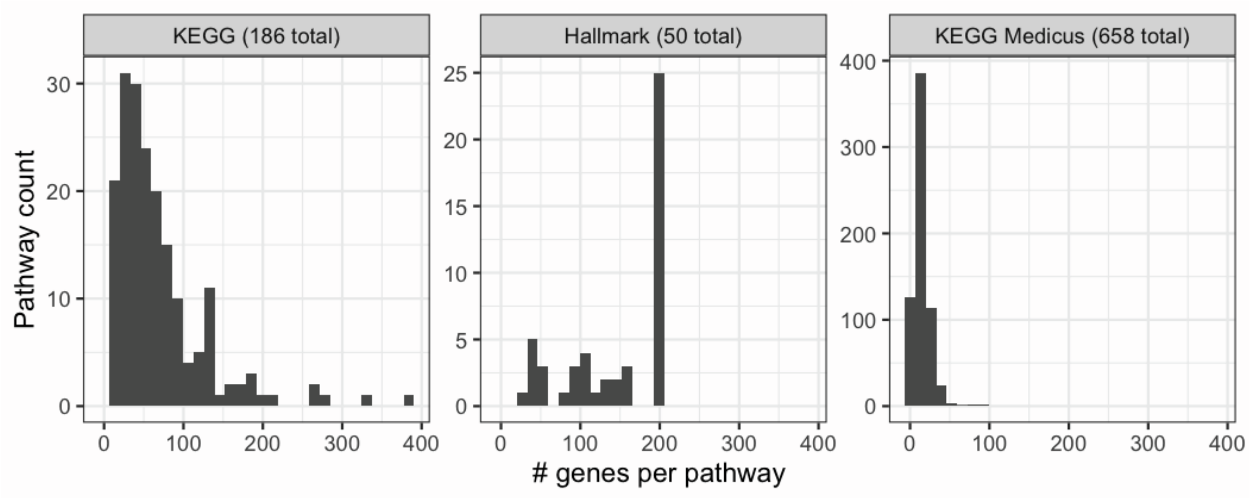

Supplementary Figure S1. Summary of selected pathway annotations from mSigDB. For each panel (corresponding to pathway groups, along with their total number of pathways), the histogram displays counts of pathways (*y*-axis) having a given number of constituent genes (*x*-axis).

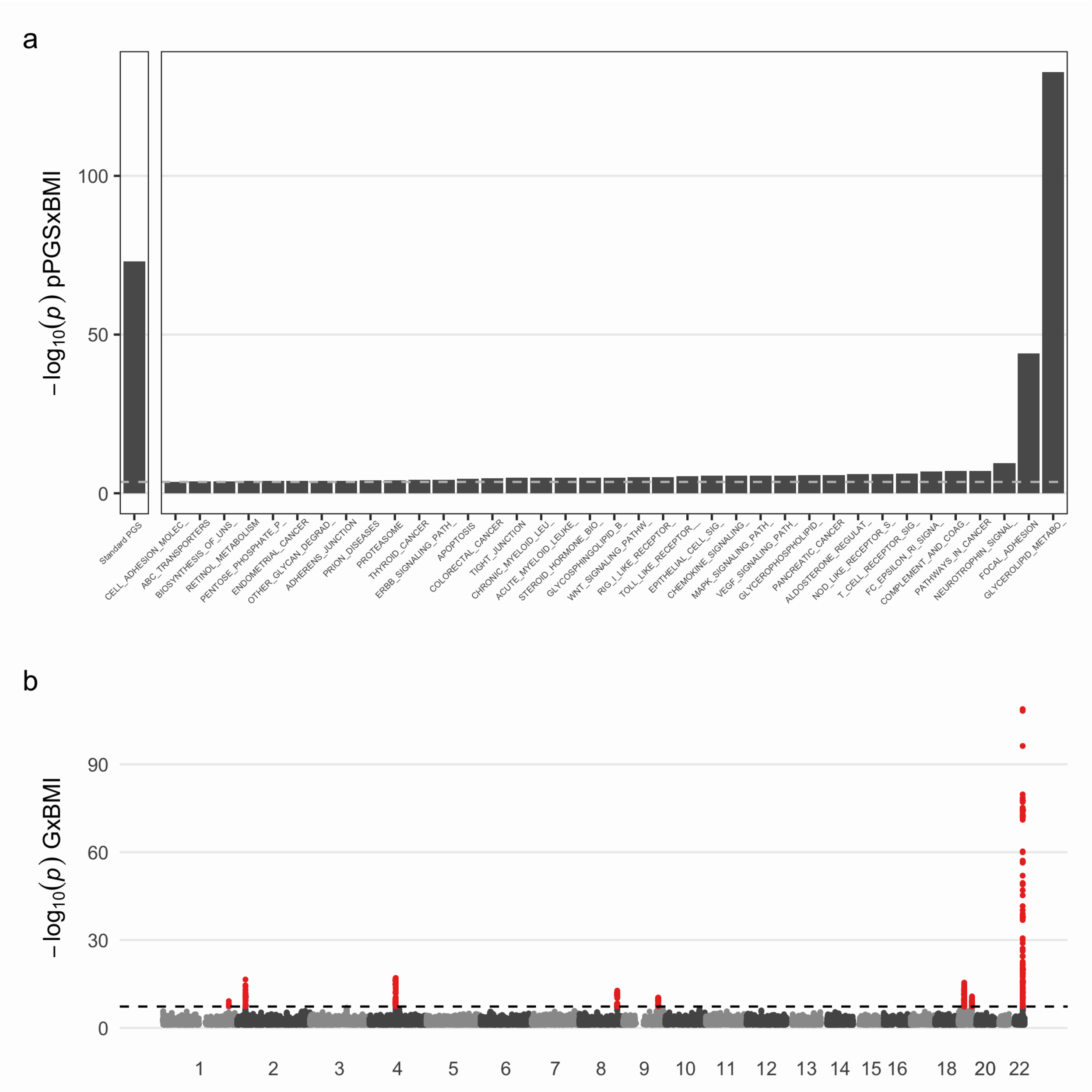

Supplementary Figure S2. GxEs shape the relationship between aspartate aminotransferase (AST) and adiposity. (a) PGSxE regression *p*-values for the gwPGS (left panel) and each significant pPGS (right panel). (b) Manhattan plot shows variant-specific *p*-values as a function of genomic position.

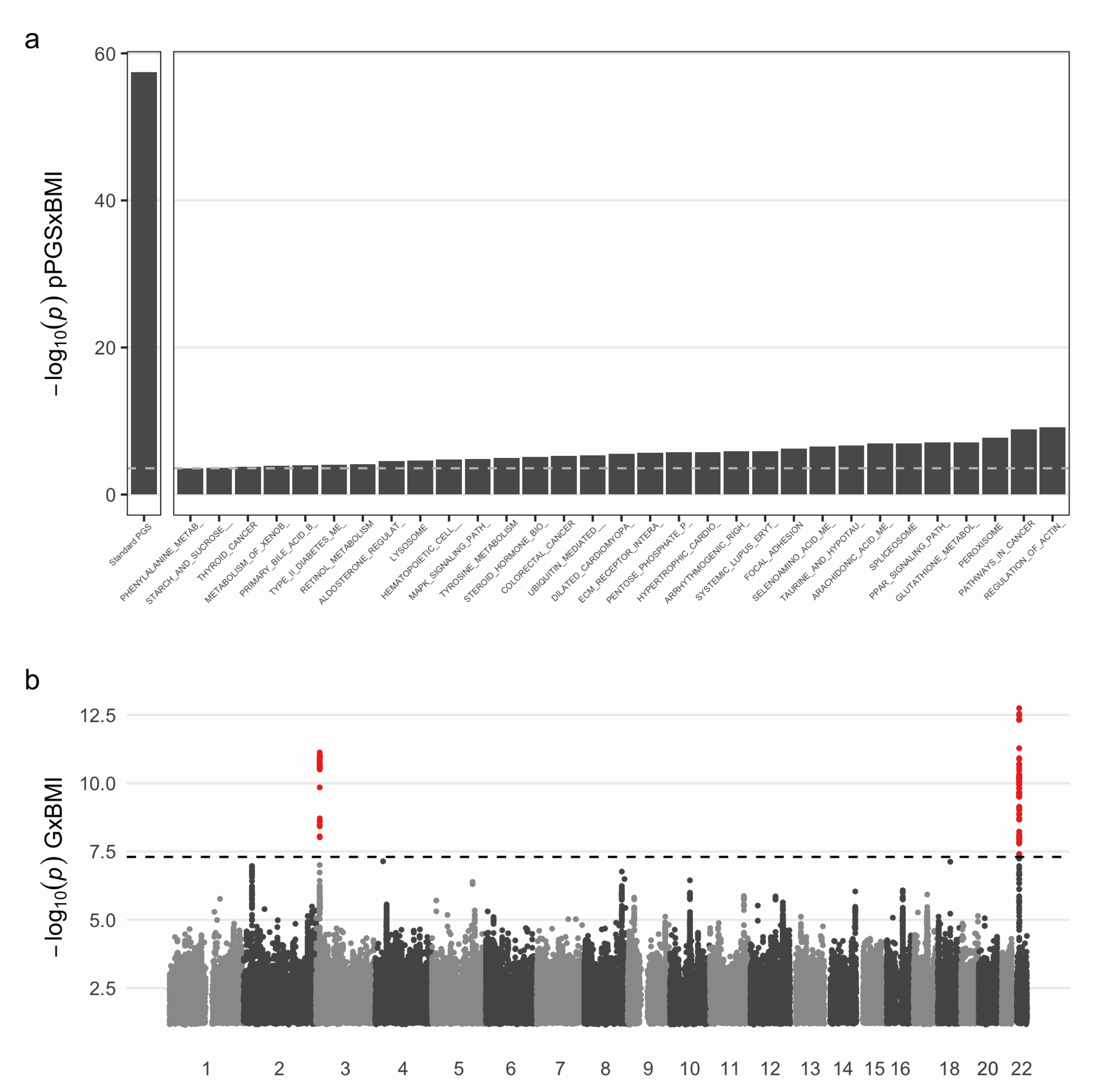

Supplementary Figure S3. GxEs shape the relationship between gamma-glutamyl transferase (GGT) and adiposity. (a) PGSxE regression *p*-values for the gwPGS (left panel) and each significant pPGS (right panel). (b) Manhattan plot shows variant-specific *p*-values as a function of genomic position.

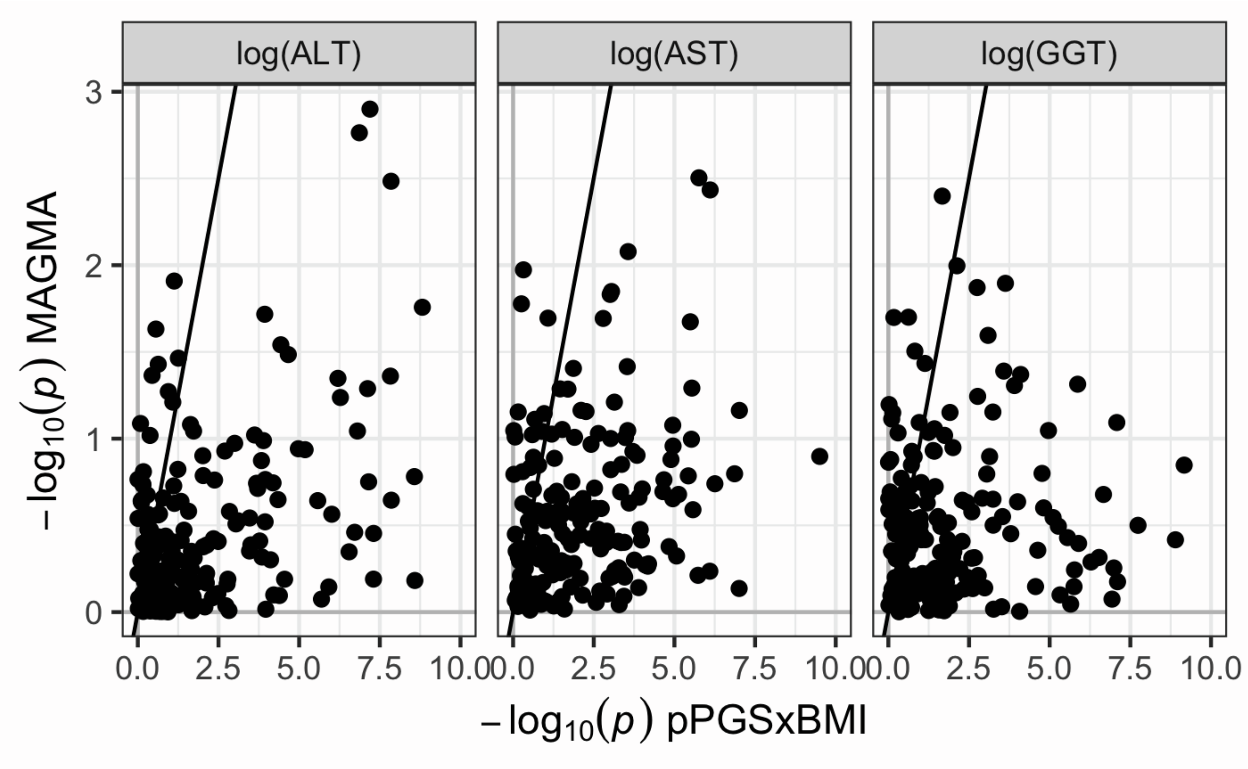

Supplementary Figure S4. Comparison of the statistical significance of pathway-level interactions tests using either pPGSxE (*x*-axis) or GWIS enrichment (based on the MAGMA tool; *y*-axis). Each point corresponds to a single KEGG legacy pathway. *x*-axis has been constrained for ALT and AST to aid visualization, which excludes results for glycerolipid metabolism and focal adhesion pathways (*p*-values reported in main text). Two points (corresponding to the “Glycerolipid Metabolism” and “Focal Adhesion” pathways) have been removed from the ALT and AST plots due to their extreme values (see Results text for their *p*-values).

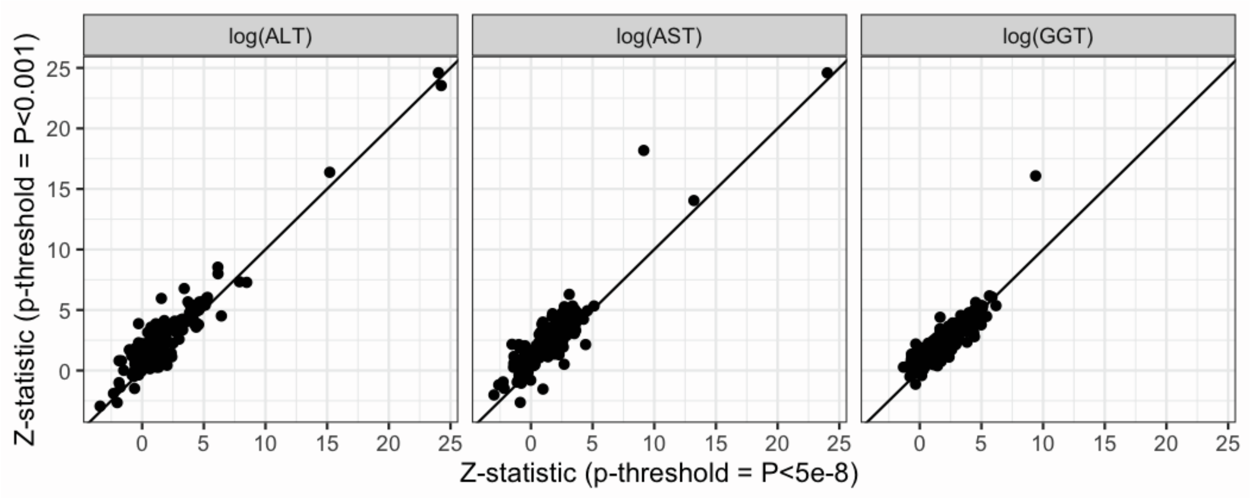

Supplementary Figure S5. Comparison of the statistical significance of pPGSxBMI tests using pPGS derived using a P&T *p*-value threshold of 0.001 (*y*-axis) versus $5x10^{-8}$ (*x*-axis). Each point corresponds to a single KEGG legacy pathway. Points could not be plotted for pathways having no annotated variants that pass $p<5x10^{-8}$.

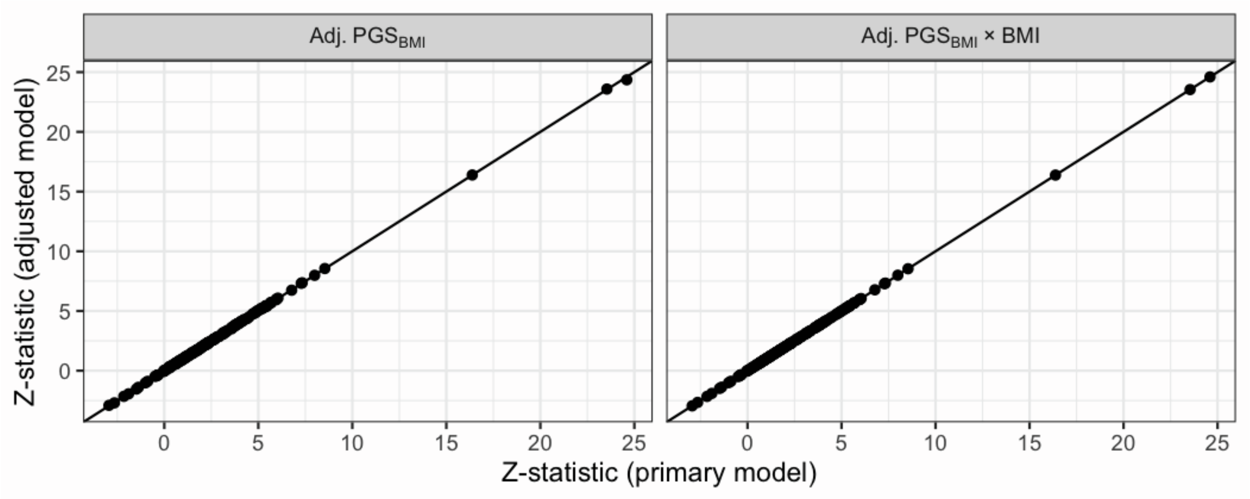

Supplementary Figure S6. Effect of PGS_{BMI} adjustment on pPGS interaction estimates. Sensitivity models adjusted for the main effect of a genome-wide PGS_{BMI} (left panel) or the main effect of PGS_{BMI} plus its interaction with physiological BMI (right panel). Interaction effect z-statistics are plotted for the primary model (*x*-axis) and adjusted model (*y*-axis).

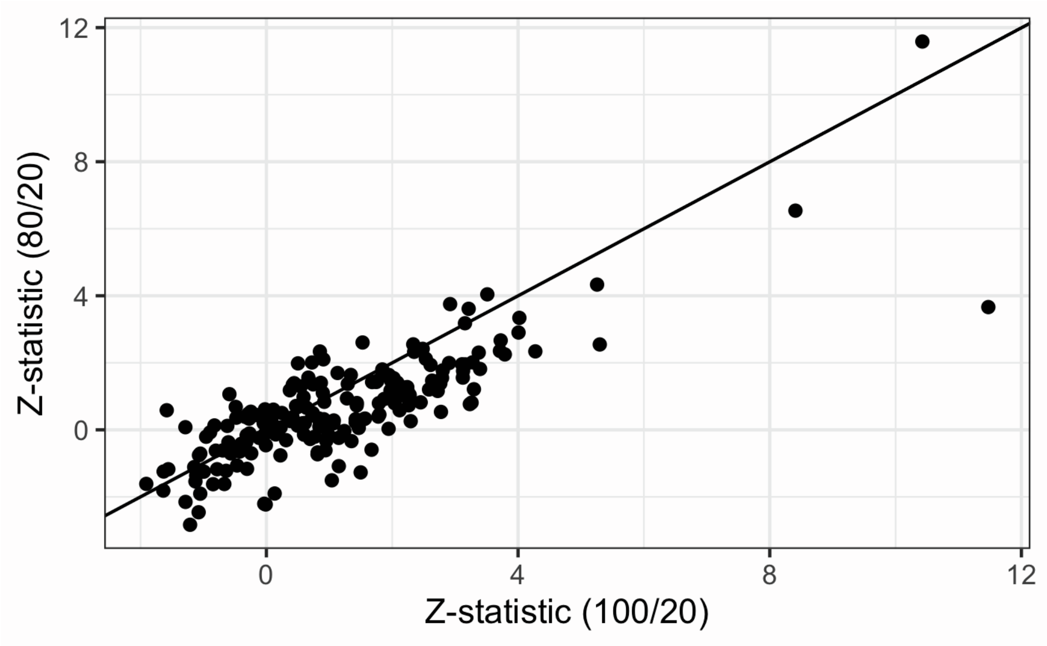

Supplementary Figure S7. Sensitivity analysis comparing primary results with those using a held-out testing set. Interaction z-statistics are compared between pPGS from the sensitivity analysis ("80/20": pPGS developed in 80% of the dataset [70% GWAS, 10% tuning] and tested in the remaining 20%) and primary analysis ("100/20": pPGS developed in 100% of the dataset and tested in the same 20%).
